# Edge and modular significance assessment in individual-specific networks

**DOI:** 10.1038/s41598-023-34759-8

**Published:** 2023-05-15

**Authors:** Federico Melograna, Zuqi Li, Gianluca Galazzo, Niels van Best, Monique Mommers, John Penders, Fabio Stella, Kristel Van Steen

**Affiliations:** 1 BIO3 - Laboratory for Systems Medicine, Department of Human Genetics, KU Leuven, Leuven, Belgium; 2 School of Nutrition and Translational Research in Metabolism (NUTRIM), Department of Medical Microbiology Infectious Diseases and Infection Prevention, Maastricht University Medical Center+, Maastricht, The Netherlands; 3 Institute of Medical Microbiology, RWTH University Hospital Aachen, RWTH University, Aachen, Germany; 4 Department of Epidemiology, Care and Public Health Research Institute (CAPHRI), Maastricht University, Maastricht, The Netherlands; 5 Care and Public Health Research Institute (CAPHRI), Maastricht University, Maastricht, The Netherlands; 6 Department of Informatics, Systems and Communication, University of Milano-Bicocca, 20126 Milan, Italy; 7 BIO3 - Laboratory for Systems Genetics, GIGA-R Medical Genomics, University of Liège, Liège, Belgium

**Keywords:** Computational biology and bioinformatics, Machine learning, Network topology, Microbial communities, Microbiome

## Abstract

Individual-specific networks, defined as networks of nodes and connecting edges that are specific to an individual, are promising tools for precision medicine. When such networks are biological, interpretation of functional modules at an individual level becomes possible. An under-investigated problem is relevance or ”significance” assessment of each individual-specific network. This paper proposes novel edge and module significance assessment procedures for weighted and unweighted individual-specific networks. Specifically, we propose a modular Cook’s distance using a method that involves iterative modeling of one edge versus all the others within a module. Two procedures assessing changes between using all individuals and using all individuals but leaving one individual out (LOO) are proposed as well (*LOO-ISN*, *MultiLOO-ISN*), relying on empirically derived edges. We compare our proposals to competitors, including adaptions of OPTICS, kNN, and Spoutlier methods, by an extensive simulation study, templated on real-life scenarios for gene co-expression and microbial interaction networks. Results show the advantages of performing modular versus edge-wise significance assessments for individual-specific networks. Furthermore, modular Cook’s distance is among the top performers across all considered simulation settings. Finally, the identification of outlying individuals regarding their individual-specific networks, is meaningful for precision medicine purposes, as confirmed by network analysis of microbiome abundance profiles.

## Introduction

When analyzing the relationship between biological features and complex traits, it is often impossible to characterize the outcome or phenotype with a single gene or a single pathway^1^, and more advanced characterizations are required. Complex diseases have no unique cause, but result from an accumulation of different and interacting variations^2^. Advances in biotechnology, such as developments in high-resolution imaging modalities and high throughput sequencing methods, have made available high-dimensional inter-dependent data on growing collections of individuals. Such data need to be analyzed robustly and stably. Network medicine allows going beyond univariate analyses and embracing the complexity of biological networks^2,3^.

Networks lend themselves well to visualizing and analyzing multiple biological processes in medicine. A network is a collection of connected objects. The objects are referred to as nodes or vertices. They are usually visualized as points. Connections between the nodes are referred to as edges or links. These are graphically drawn as lines between points. Such networks may be appended with extra information, such as node labels or edge weights. A module is a subnetwork composed of a subset of selected nodes and edges. Network modularity measures the strength of division of a network into modules. More details are in Table S1. Graph-theoretical constructs such as modules may be more robust and effective than traditional clinical variables in predictive or descriptive models^4^. They are often compared between graphs, where each graph may represent a different condition or state (f.i. diseased versus healthy). As we will see later, networks may also be constructed for each individual separately.

Population-based biological models, which infer edges in biological networks by pooling samples together or fixing a unique network wiring applicable to all individuals in a target group, have been used to extract features for downstream informed analyses^5^ or to guide epistasis detection and interpretation using genome-wide association study designs^6^. From the lens of personalized medicine, they have also been shown to help draw patient-specific conclusions (e.g.,^7^). However, a “one size fits all” medicine is no longer acceptable^8,9^, and extrapolating conclusions from population-derived networks may not be specific enough for a particular individual. Furthermore, whereas statistical interactions occur at a population level, biological interactions occur at an individual level^10^. Thus, considering that biologically relevant interactomes may vary from one individual to another, constructing individual-specific networks with individual-specific edges has received growing interest.

Here, we define an individual-specific network (ISN) as a network describing a single individual, with edges (edge weights) that may differ between individuals. As a consequence, comparing ISNs implies comparing potentially different network wirings. Examples of ISNs that fit this definition are the *differential networks* of^11,12^ and the *completed networks* of^13–15^. In differential networks, individual-specific edge weights are obtained by contrasting population-based edge weights between the entire population and the population with the individual added or removed. Hence, edges harbour information about an individual’s influence on a population. In completed networks, each ISN is standalone and assumes an individual comes from a distribution with the population-based reference network as the expected network. Investigating new methods of measuring variation, such as via individual-specific edges and modules, can provide a different perspective on analyzing existing data, to improve endotype identification, risk prediction, and treatment planning.

Individual-specific networks are not a new concept. In principle, once we have enough information about an individual, taken over time or under multiple conditions, we can exploit the multiplicity and build a network that is unique to that individual. Several examples link to neurosciences^16–19^. Others link to functional networks between cells (for instance, reflecting the positions of beta-cells in tissue slices^20^). However, quite often, the collected data are static or pertain to a single condition. Hence, one of the challenges of ISNs includes their construction in the absence of repeated measures over time or conditions. The first edge-inference approaches in this sense were discussed and developed in^21^ and^13^ and depend on selecting a reference population, adding or removing an individual, and re-estimating the network with the augmented or reduced population, respectively. Another challenge is how to extract relevant information from a derived ISN. Common practice is to aggregate information, such as averaging edge weights in each ISN, and then look for associations with phenotypes of interest (for instance, drug reaction and time-to-clinical-event^22,23^). The most common objective of studies that include ISNs as input is prediction (for a review, see^24^). This usually involves extracting graph-theoretical features and linking them to a phenotype of interest. Unfortunately, doing so may dilute the full potential ISNs bring about^25^. The primary challenge is often poorly addressed: for which individuals is it essential to construct and interpret an ISN?

In this work, we take the challenge of assessing whether a constructed individual-specific network significantly differs from a population-based network while embracing network complexity beyond edges. We do so by formulating the challenge as an outlier detection problem (i.e., the problem of finding patterns in data that do not agree with expected behavior). We focus on the ISNs of Kuijjer^13^, defined in the II. subsection of the “Methods” section when developing and evaluating edge and modular significance assessment strategies. These networks are hereafter referred to as *ISNs-L* (short for LIONESS, the name of Kuijjer’s ISNs approach). A necessary intermediate step for *ISNs-L* calculation is the network derived from a reference population by removing one individual, which we call *LOO* network. There are many advantages of *ISNs-L* networks. Cardinally, it allows the translation of network interpretation strategies from population to individual; it also empowers focusing on each individual and his/her specific dynamics and associations; lastly, it departs from the notion of a network derived from a collection of individuals that can be seen as a model for an average individual. Moreover, for completion we compare the results obtained on *ISNs-L* with results on another ISN approach: SSN (sample-specific network)^21^.

Our work overcomes the limitations of current practices with ISNs. The major limitation is that the significance assessment of an ISN usually relies on large-sample statistics that involve highly correlated samples (only differing from each other by a single sample). As a result, evaluating the statistical significance of *ISNs-L* and, in this way, identifying extreme or exceptional individuals remains an under-investigated problem. Furthermore, significance assessment is, at best, verified on a per-edge basis. Popular examples involve differential networks developed in^14,21,26^. Single-edge significance assessments have reported limitations^27^. Edges may not occur in total isolation but in a strongly connected and interdependent ecosystem imposed by the whole network. Both from an analytical or translational point of view, modules may therefore be more suitable instruments when assessing the statistical significance of an individual through its ISN. To the best of our knowledge, no formal report exists about module significance assessment in the context of ISN outlier detection.

The main contributions of this work are as follows: (i) development of novel methods for outlier detection, particularly a modified modular *Cook’s distance* measure and leave-one-out methods (*LOO-ISN* and *MultiLOO-ISN*); (ii) customization of existing outlier detection methods *kNN*, *OPTICS*, and *Spoutlier* to accommodate ISNs; (iii) introduction and assessment of the relevance of a novel modular significance assessment paradigm with ISNs; (iv) evaluation via synthetic data and validation via real-world data while assessing strengths and weaknesses of edge-oriented and module-oriented considered strategies. This article addresses the literature gap by developing a measure of significance for ISNs that enables deciding which individuals would benefit from individual-specific network analysis.

The paper is organized as follows. We divide the “Results” section into three subsections: two extensive simulation studies with different distributional assumptions and a microbiome data application. Hyperparameters are allowed to vary according to a grid of choices. The “Discussion” section presents main insights and suggests new research questions. In the “Methods” section, we describe data and methodologies. Further details are presented as Supplementary Material. A glossary of terminology is provided in Supplementary Table S1.

## Results

The performance of the proposed outlier detection methods is evaluated and compared on both synthetic and real-world data. Our real-life use case is a human microbiome study. The synthetic data reflect two scenarios: one with gene expression and one with microbial profiles available for a population of individuals. These two scenarios imply different underlying distributions to generate the data, with gene expressions assumed to be normally distributed and for microbial data respecting the compositional nature of the data. In synthetic data, outlier and non-outlier individuals are sampled from two different distribution, each one using different parameter values, i.e., a different variance/covariance matrix, quantifying the associations between variables; thus the ground truth, i.e., whether an individual is an outlier (1) or not (0), is known. On the simulated dataset of analysis (dimension: $N \times k$, with *N* individuals and *k* variables), we calculated Pearson correlation to create the population-based network (dimension $k \times k$). On the population-based network, we calculated the ISN for each individual. Said ISNs constitute the input for the proposed outlier detection methods, with the individual-specific edge weights being the feature set. The various steps are illustrated in Fig. S1. Hence, for each individual, its’ ground truth is confronted with the ranked outlier score computed by each method. The outlier score (OS) for a certain individual is the degree to which a certain method classifies the individual as an outlier. The comparison of the effectiveness of different methods is performed under different experimental conditions and using a given grid of hyperparameters values. As a real-world case study, we considered a portion of the LucKi cohort^28^ with infant microbiomes collected over time. Exploring methods to identify meaningful modules in a network is a broad field that exceeds the scope of this paper. The proposed methods are agnostic to the chosen module detection algorithm. For the real-world case study, we used the SPINGLASS^29^ algorithm to identify modules.

Methods evaluated and compared in this paper belong to one of the following groups: (i) novel proposals, (ii) adaptations of existing methods, and (iii) scientific literature’s methods. Out of the scientific literature’s methods, only *SSN*^21^ has been previously reported in the ISNs field. Given that Liu^21^ introduces a significance assessment method and a network construction technique, both usually referred to as *SSN*, we will refer to them respectively as *SSN-m* and *SSN-n*. Furthermore, depending on their rationale, methods are grouped into the following *families*; (i) leave-one-out, (ii) Cook’s distance, (iii) Spoutlier, and (iv) kNN and OPTICS. The leave-one-out (*LOO*) family exploits the impact of removing one individual at a time from the dataset of analysis; it includes; (i) *LOO-ISN*, (ii) *MultiLOO-ISN*, and (iii) *SSN-m*. The Cook’s distance family is a collection of modular Cook’s distance aggregations, including our proposals referred to as; *Cook’s med*, *Cook’s max*, and *Cook’s mean*, which differ by the adopted aggregating function, i.e., respectively median, maximum and mean. An iterative procedure calculates Cook’s distances. The algorithm considers an edge as the target and predicts its’ value (edge weight) via all the other edges belonging to the given module. The Spoutlier family originates from Sugiyama’s^30^ work and employs a fixed reference set in nearest neighbours. We refer to the original implementation as *Spoutlier-l*. The adaptations of Spoutlier methods are referred to as *OTS* and revolve around alternative distance measures, reference set computations, and ensembling. *OTS euclidean* and *OTS cosine* employ euclidean distance and cosine dissimilarity, respectively, and both use a modified reference set than *Spoutlier-l*. *MOTS euc* and *mOTS cosine* are an *ensemble* on *OTS euclidean* and *OTS cosine*, respectively. Finally, *mOTS glob* employs both *OTS euclidean* and *OTS cosine* as base predictors.

To the best of our knowledge, methods belonging to the kNN^31^ and OPTICS^32^ family have never been applied in the ISNs field. For each method, we explored multiple hyperparameter values. In the *kNN* family, *kNN 5*,$\sqrt{N}$ with the parameters $k_{min}$ and $k_{max}$ set to 5 and $\sqrt{N}$ achieves the best simulation performances, and it is therefore referred to as *kNN*. A thorough description of every method and parameters’ settings can be found in the *methods* section, along with a comprehensive Table S2 containing every acronym’s characteristic in the Supplementary section. The aforementioned methods are applied to *ISNs-L*, but the same numerical experiments have been performed on *SSN-n* for comparison purposes. Results from the application of the *SSN-n* methods are identified with the suffix *-n*.

Lastly, numerical experiments based on synthetic data have been evaluated by comparing the calculated outlier score *OS* to the ground truth *GT*, and thus by constructing a *ROC* curve. The area under the curve *AUC* is used as a performance measure.

### Synthetic data: normally distributed

This simulation scheme aims to mimic gene co-expression networks. More details on the characteristics of gene co-expression networks are provided in Supplementary Table S1. We formed an *experimental grid* by generating synthetic data for different values of the following parameters; sample size *N*, module’s size *k*, number of outliers *M*, and probability distribution that generates outliers (more details in Section “Synthetic data” of the “Methods”). Each entry (row) of the experimental grid is referred to as a *setting* consisting of 200 *runs*. Each run outputs a dataset whose rows are associated with individuals and whose columns are associated with variables (nodes). Furthermore, each row is associated with a binary variable, the ground truth, which tells whether an individual is an outlier or not.

The dataset is used to calculate the population-based network (dimension $k\times k$), with its base element being the association between nodes $v_i$ and $v_j$. Said population-based network characterizes the associations (in our work, Pearson correlation) between the variables and defines the adjacency matrix. From the population-based network, an individual network (*ISNs-L* or *SSN-n*^21^) is computed to be the downstream analysis input. The set of individual-specific edge weights in a module constitutes the feature set of the outlier detection methods.

A *realization* is defined as the result of applying a method to a setting; for each realization, the *OS* is computed for each individual-specific network, quantifying the support for the individual to be an outlier. Hence, these score values can be ranked to find those individuals most likely to be outliers. Then, for each method and for each setting, we summarize the results of the corresponding 200 runs with the *Median* *AUC* due to its robustness to extreme values. As a coarse summarization, we average across all settings, calculating *Mean* and *Median* *AUC* values for each method. These scores are reported in Table 1.

*Cook’s med* achieves the best *Median AUC* value (0.920), while *mOTS cosine* achieves the best value of *Mean AUC* (0.866). The OPTICS methods are not effective, achieving performance values that are barely better than a random guess. Finally, neither kNN nor *mOTS euc* achieve an aggregate *AUC* value greater than 0.7, while all leave-one-out methods (MultiLOO-ISN, LOO-ISN, SSN-m) achieve aggregate *AUC* values smaller than 0.64. As explained in detail in the *method* section, not all methods apply to every setting, so the comparison is incomplete. For clarity, only the top methods for each family, in terms of *AUC*, are reported in Table 1. A comprehensive Table S3 is available in the Supplementary.

**Table 1:** Synthetic data: normally distributed: averaged *AUC* values achieved by different methods.

Method	Median AUC	Mean AUC
MultiLOO-ISN	0.628	0.632
LOO-ISN	0.582	0.591
SSN-m	0.584	0.601
KNN log(N),P	0.646	0.657
KNN 5,$\sqrt{N}$	0.649	0.659
Optics 5	0.608	0.595
Optics $\sqrt{N}$	0.532	0.529
OTS euclidean	0.628	0.637
OTS cosine	0.812	0.773
mOTS cosine	0.880	**0.866**
mOTS euc	0.629	0.640
mOTS glob	0.820	0.824
Spoutlier-l	0.628	0.639
Cook’s max	0.903	0.853
Cook’s med	**0.920**	0.859

Bold values indicate the top performer of each column.

The Cook’s med method achieves the best *Median AUC* value, while the mOTS cosine method achieves the best *Mean AUC* value.

#### Results by sample size *N*

**Figure 1:**
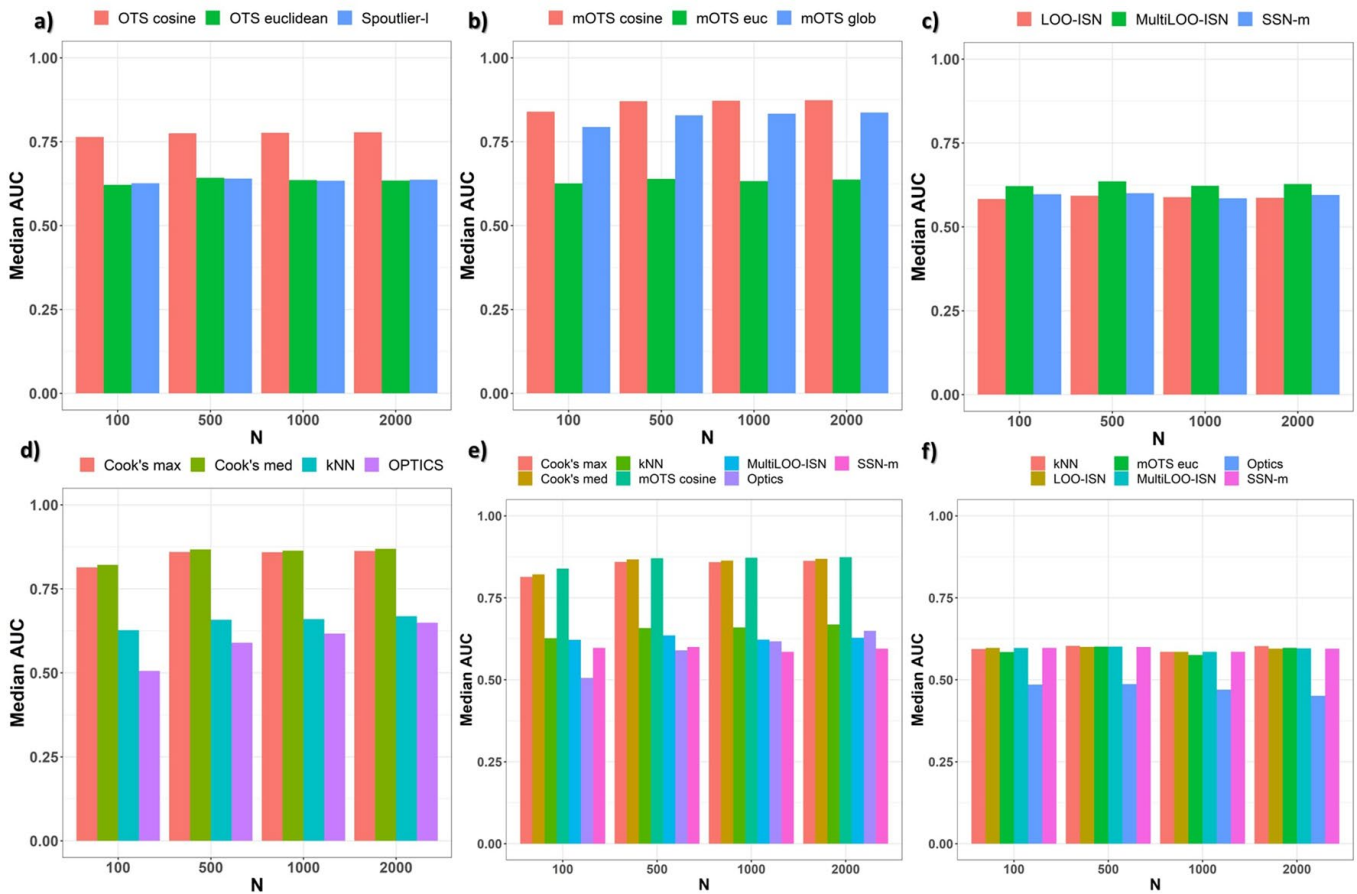
Synthetic Data: normally distributed. *AUC* values of various methods. (**a**) single-shot Spoutlier methods are compared. *OTS cosine* evenly dominates over the canonical *OTS euclidean*. (**b**) the ensemble methods are compared, and *mOTS cosine* is the best for all values of the sample size *N*. (**c**) p-value yielding methods are compared, and *MultiLOO-ISN* outperforms the counterparts. (**d**) the remaining methods are compared, with *Cook’s med* consistently dominating for all values of the sample size *N*. In the bottom panel, selected methods are compared. *e*) the comparison comprehends all settings: *mOTS cosine* and Cook’s methods (both *Cook’s med * and *Cook’s max*) consistently dominate their counterparts. *f*) the comparison is restricted to single-edge ($k=2$) settings: no method achieves an *AUC* value greater than 0.7.

This section compares different methods in terms of the achieved performance when grouping the sampled synthetic data by sample size *N* = $\{100,500,1000,2000\}$. By comparing different Spoutlier’s implementations in single-shot (i.e., the method applied once, no ensemble), *OTS cosine* performs overly better than *OTS euclidean*. As highlighted in Fig. 1a,b, *OTS* and *mOTS cosine* achieve an *AUC* value ranging from 0.75 to 0.90. Euclidean counterparts achieve an *AUC* value lower than 0.65. At the same time, no difference is detected between the literature’s *Spoutlier-l* and the custom *OTS euclidean* approach: introduced reference set computation performs no better nor worse than the literature’s one. The proposed ensemble implementations achieve better results than their single-shot counterparts. Considering the median of the *OTS predictions* over all the repetitions is highly effective. *mOTS cosine* is the best Spoutlier method, achieving an *AUC* value greater than 0.8 for every value of *N*.

Methods yielding p-values, i.e., *LOO-ISN*, *MultiLOO-ISN* and *SSN-m*, represent a relevant facet of the current study, providing a clear threshold to detect outliers. A comparison between these methods is depicted in Fig. 1c, and shows that *MultiLOO-ISN* outperforms *LOO-ISN* for all values of the sample size *N*. Notably, for the literature’s method *SSN-m* only single-edge ($k=2$) comparison is possible, thus only those cases are depicted. *kNN* and *OPTICS* never achieve *AUC*$> 0.7$ (Fig. 1d). Furthermore, the best methods for each family are shown together to get a glimpse of their performance under different sample size values, Fig. 1e. Cook’s distance and *mOTS cosine* stand out, achieving *AUC* values greater than 0.8 for all size values *N*. These methods dominate their corresponding counterparts by more than 0.2 for each setting. No method achieves an acceptable performance value, i.e., *AUC*$> 0.7$, for single-edge settings (Fig. 1f), thus highlighting the need for modular assessments. Finally, we notice a slightly positive association between *AUC* and sample size *N*.

#### Results by module’s size *k*

**Figure 2:**
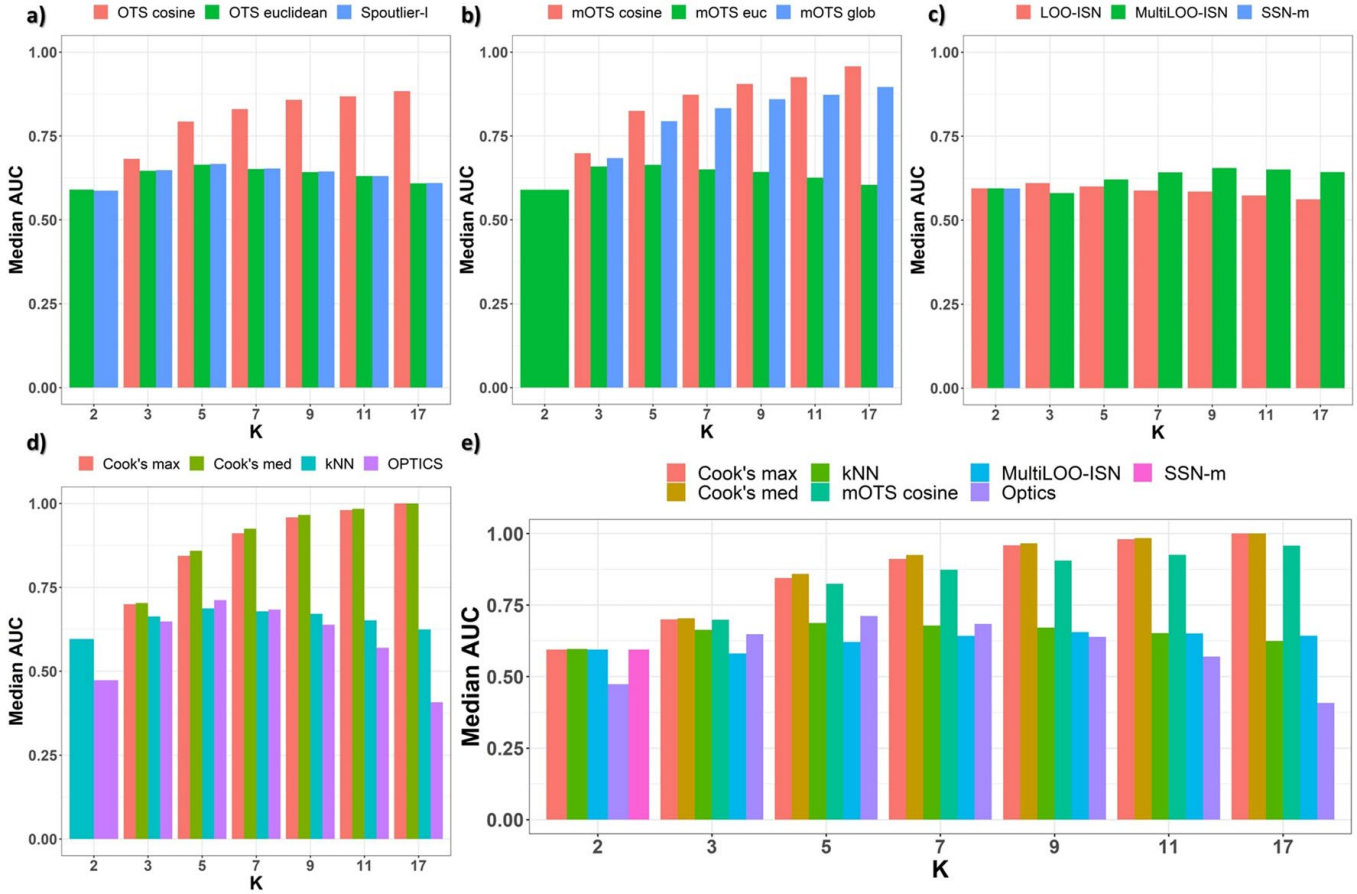
Synthetic data: normally distributed. *Median* *AUC* on the *y* axis and module’s size *k* on the *x* axis. (**a**) single-shot Spoutlier’s methods are compared. *OTS* cosine evenly dominates its’ euclidean counterpart (*OTS* euclidean) for $k>2$. (**b**) the ensemble methods of the Spoutlier family are compared, and *mOTS cosine* is the best for $k>2$. (**c**) p-value yielding methods are compared, and *MultiLOO-ISN* achieves the best performance for $k\ge 5$. (**d**) remaining methods are compared, with *Cook’s med* consistently dominating all others when $k>2$. at the bottom panel, selected methods are compared together. (**e**) the comparison includes all settings: cosine-based *OTS* and Cook’s distance methods consistently dominate their counterparts when $k>2$. No method achieves satisfactory performance under the $k=2$ settings.

In modular settings ($k>2$), the adapted Cook’s distances methods, i.e., *Cook’s med* and *Cook’s max*, achieve the best values of performance. They are closely followed by the *mOTS cosine* method. By grouping the synthetic data per module’s size $k= \{2,3,5,7,9,11,17 \}$, a positive relationship between the module’s size *k* and performance *AUC* emerges in *(m)OTS cosine* (Fig. 2a,b) and Cook’s distances methods (Fig. 2d,e). Other methods (Fig. 2c) do not show an association with the module’s size *k*. Crucially, no method achieves a satisfactory performance value in the single-edge analysis setting: when *k = 2*, every method achieves an *AUC* value smaller than 0.6. The limited informativeness of an edge alone emerges from those results. Other noteworthy insights originate from comparing Spoutlier’s methods (Fig. 2a,b). *mOTS* euclidean is upper-bounded by 0.7, while mOTS cosine achieves an *AUC* value greater than 0.9 for large module sizes *k*. *mOTS glob*’s *AUC* is positively associated with the module’s size *k* and, coarsely, around 0.05 worse than *mOTS cosine*. *mOTS glob*’s performance, although suboptimal, hints toward the value of combining both an arithmetical and a geometrical point of view. The scenario is a carbon copy of the single-shot setting: *OTS cosine* is positively associated with the module’s size *k*, and results are more than 0.2 better than the *OTS euclidean* counterpart for high values of *k*.

#### Results by number of outliers *M*

Figure S2 shows that the number of outliers does not affect the behaviour of *AUC* with respect to the module’s size *k*. When changing the number of outliers *M* = $\{1, 5, 10\}$, the relative ranking of methods appears to be stable. Furthermore, the slope between performance *AUC* and the module’s size *k* does not change. The performance, i.e., *Median* *AUC* over all runs, achieved by the best methods, is negatively associated with the number of outliers *M*: when more individuals are outlying, the performance decreases. Furthermore, no interaction emerges between the number of outliers *M* and the module’s size *k*. Cook’s distances methods, i.e., *Cook’s max* and *Cook’s med*, dominate other methods regardless of the number of outliers *M*. These methods also exhibit robustness for the number of outliers *M*, by achieving a stable performance value in all settings, with specific reference to those cases where the module’s size *k* is large.

### Synthetic data: compositional

This simulation scheme aims to mimic microbial co-occurrence networks, thoroughly described in Supplementary Table S1. In this section, we present and discuss the performance achieved by methods on a microbial simulation scheme. A subset of the grid used to simulate normally distributed data is combined with a grid explicitly designed for compositional simulations, thus increasing the computational burden. In detail, additional parameters are (i) *Data heterogeneity*, (ii) *Multiplying factor* (*Mult*) multiplier to differentiate each individual’s variable (node) set between outliers (also referred to as *cases*) and non-outliers (*controls*), and (iii) the ratio of inflated taxa to the total. Therefore, we develop a parallel implementation where multiple instances of the same simulation setting, with different *random starts*, have been performed. The overall experimental plan accounts for 150 runs for each setting included in the augmented parameter’s grid (in total, 972 settings).

Then, following the same procedure as in the previous section, we create the *ISNs-L* and *SSN-n* networks for downstream analysis. For each realization, the corresponding *AUC* value is computed and averaged over all 150 runs to obtain the *Median AUC* value achieved by each method.

We identify the level of *Data heterogeneity* and the *Multiplying factor* as primary performance drivers. Hence, we average over all settings grouped by primary performance’s driver, i.e., *Data heterogeneity* and *Multiplying factor*, calculating *Mean* and * Median* *AUC* values for each method. Table 2 reports on *Mean* and *Median* *AUC* values of selected methods under different *Data heterogeneity* and *Multiplying factor* settings. We refer the interested reader to the Supplementary for an in-depth analysis.

Performances achieved by various methods positively correlate with both *Data heterogeneity* and *Multiplying factor*. We achieve the best values of *AUC*s when *Mult = 2* and *Data heterogeneity = high* (Table 3). The *kNN* and the *LOO-ISN* methods are consistently among the best-performing methods for all settings. The euclidean-based methods *OTS*, i.e., both *OTS * and *mOTS euclidean*, together with Cook’s distance methods, are competitive. Furthermore, no method achieves an *AUC* $>0.51$ in settings where *Mult = 1.1*. Under this scenario, the discrepancy between *cases* and *controls* is feeble.

We highlight the main differences between considered methods by focusing on settings with *high* heterogeneity and *high* multiplier, i.e., high differentiation between taxa in *cases*’ and *controls*’ individuals. *kNN* achieves the best *Mean* (0.801) and *Median* (0.803) AUC values. Among the best, achieving both *Mean* and *Median* AUC values greater than 0.77, we find *LOO-ISN*, *Spoutlier-L*, *Cook’s max*, *OTS* euclidean, and *mOTS* euc. Cosine-similarity-based methods *OTS* achieve low *AUC* values and do not seem suited to accomplish the task. Furthermore, we observe that different choices for methods’ parameters, except the distance measure in the case of *OTS*, have feeble or no influence on the final performance.

**Table 2:** Synthetic data: compositional.

Method	MULTIPLIER 1.1		MULTIPLIER 1.5		MULTIPLIER 2.0		UNIFORM—NO HET		PARETO—4 MILD HET		PARETO—0.7 HIGH HET	
	Median AUC	Mean AUC	Median AUC	Mean AUC	Median AUC	Mean AUC	Median AUC	Mean AUC	Median AUC	Mean AUC	Median AUC	Mean AUC
MultiLOO-ISN	0.502	0.502	0.565	0.586	0.684	0.705	0.543	0.562	0.556	0.578	0.629	0.653
LOO-ISN	0.503	**0.505**	**0.586**	**0.598**	**0.726**	**0.733**	**0.559**	**0.585**	**0.571**	**0.601**	0.637	0.650
SSN - m	0.498	0.497	0.559	0.577	0.676	0.695	0.541	0.562	0.553	0.575	0.619	0.632
KNN log(N),P	0.503	0.503	0.582	0.596	0.714	0.730	0.556	0.579	0.567	0.595	0.642	0.656
KNN 5,$\sqrt{N}$	**0.504**	0.502	0.582	0.597	0.717	0.732	0.556	0.579	0.568	0.595	**0.644**	**0.657**
Optics 5	0.494	0.477	0.541	0.533	0.633	0.624	0.510	0.525	0.521	0.536	0.546	0.573
Optics $\sqrt{N}$	0.491	0.489	0.556	0.565	0.673	0.686	0.532	0.555	0.545	0.569	0.595	0.616
OTS euclidean	0.503	0.503	0.577	0.591	0.704	0.718	0.553	0.574	0.566	0.590	0.635	0.648
OTS cosine	0.499	0.500	0.504	0.504	0.499	0.503	0.498	0.497	0.496	0.495	0.509	0.514
mOTS cosine	0.499	0.499	0.503	0.506	0.497	0.505	0.494	0.493	0.491	0.491	0.519	0.525
mOTS euc	**0.504**	0.503	0.581	0.595	0.714	0.729	0.554	0.578	0.569	0.595	0.637	0.655
mOTS glob	0.502	0.503	0.551	0.571	0.642	0.672	0.532	0.550	0.547	0.564	0.614	0.631
Spoutlier -l	0.503	0.501	0.576	0.590	0.703	0.720	0.551	0.575	0.561	0.589	0.634	0.646
Cook’s max	0.501	0.502	0.575	0.594	0.703	0.720	0.554	0.574	0.564	0.589	0.637	0.652
Cook’s med	0.502	0.503	0.580	0.593	0.712	0.718	0.555	0.578	0.566	0.592	0.630	0.644

Bold values indicate the top performer of each column.

Summarization of methods’ *Median* and *Mean* performances per *Mult* parameter—if the average abundances for the *cases* individual are $10\%$, $50\%$ or $100\%$ more - and *Data* *Heterogeneity*—from no to mild and high. High multipliers and more heterogeneity yield better *AUC*. With a *Mult* of 1.1, outlier detection is not better than random guessing. Notably, there is an appreciable performance gain passing from mild to elevate heterogeneity, but the difference from no to mild heterogeneity is limited. *KNN*’s methods and *LOO-ISN* are consistently among the best in every scenario, with *Cook’s distance* and euclidean-based *Spoutlier* methods closely following. Furthermore, cosine *OTS*, both *OTS cosine* and *mOTS cosine*, have worse performance than their euclidean counterparts. *LOO-ISN* achieves the top performance, 0.726, in terms of *Median AUC*, in the $Mult=2.0$ scenario. This method has a 0.221 performance increment from $Mult=1.1$ to $Mult=2.0$, highlighting the multiplier as the primary driver of performance.

**Table 3:** Synthetic data: compositional.

Mult 2 & Pareto—0.7		
Method	Median AUC	Mean AUC
MultiLOO-ISN	0.780	0.794
LOO-ISN	0.788	0.788
SSN m	0.758	0.760
KNN log(N),P	0.800	0.800
KNN 5,$\sqrt{N}$	**0.801**	**0.803**
Optics 5	0.686	0.669
Optics $\sqrt{N}$	0.739	0.74
OTS euclidean	0.786	0.786
OTS cosine	0.515	0.519
mOTS cosine	0.542	0.544
mOTS euc	0.800	0.799
mOTS glob	0.739	0.754
Spoutlier -l	0.789	0.787
Cook’s max	0.786	0.793
Cook’s med	0.775	0.776

Bold values indicate the top performer of each column.

Averaged *AUC* in the context of *high* heterogeneity and *elevate* multiplier in synthetic data. KNN methods achieve the best performance, with *KNN 5,* $\sqrt{N}$ yielding *Mean AUC* $=0.803$ and *Median AUC* = 0.801, and *kNN log(N),P* closely following. *Euclidean* Spoutlier, i.e., *Spoutlier-l, OTS euclidean * and *mOTS euc*, Cook’s distance methods, i.e., *Cook’s max* and *Cook’s med*, and *LOO-ISN* are also strong performer, all with *Median AUC* $\ge 0.77$. Cosine OTSs methods are not suited for the task and barely better than a random guess.

#### Results by module size *k*

**Figure 3:**
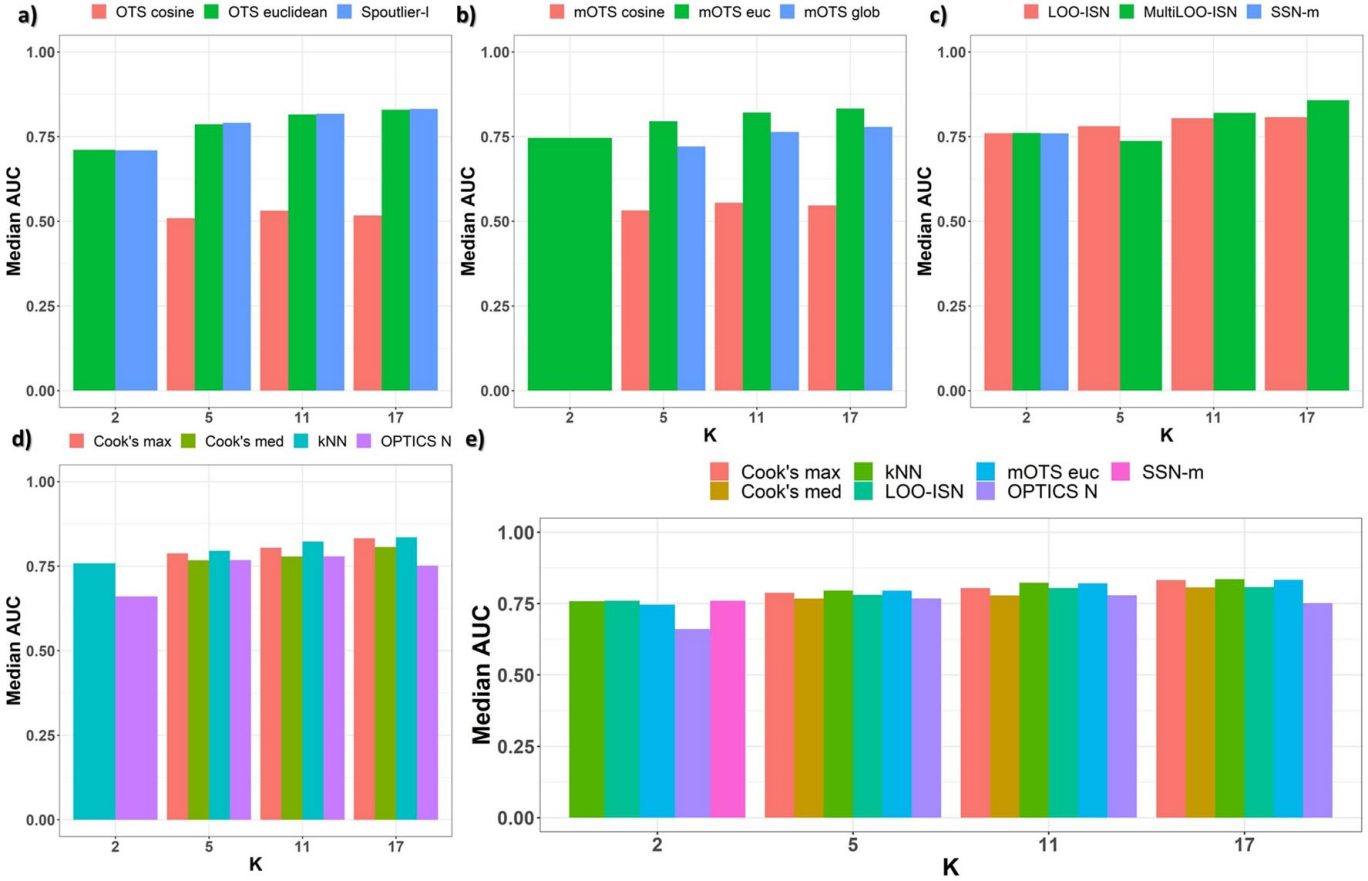
Synthetic data: compositional. Median *AUC* on the *y* axis and module’s size *k* on the *x* axis. (**a**) single-shot Spoutlier methods are compared. Euclidean methods, both *OTS* euclidean and *Spoutlier-l*, dominate *OTS cosine*. (**b**) Spoutlier’s ensemble methods are compared, and *mOTS euc* uniformly emerges as the best Spoutlier implementation when $k>2$. (**c**) p-value yielding methods are compared, and *MultiLOO-ISN* achieves the best performance starting for modular settings, i.e., $k>5$. On (**d**), the remaining methods are compared, with *kNN* and *Cook’s max* consistently dominating their’ counterparts. In the bottom panel, selected methods are compared together. On (**e**), the comparison includes all settings: *KNN*, *mOTS euc*, *LOO-ISN*, *Cook’s max* and *Cook’s med* consistently achieve good performance.

Here, we analyze performances when grouping simulation runs by module’s size *k* = $\{2,5,11,17\}$. Given the considerable heterogeneity in the data analyzed, the focus is set on the aggregation of iterations in settings where $Mult=2$ and the *Heterogeneity level* is *high*. Notably, *LOO-ISN* performs better than *MultiLOO-ISN* for $k<5$, while *OTS euclidean* performs better than its’ *cosine-based* counterparts, in contrast to results for simulations under the normality assumption. A mild positive association between the module’s size *k* and performance (median *AUC*) is observed in Fig. 3, thus highlighting the inner modularity nature of those estimates.

Unlike what we observed for simulations under the normality assumption, the assessment is informative for single-edge settings: the median *AUC* is around 0.75 for most of the considered methods. Other noteworthy results originate from comparing Spoutlier methods. Indeed, euclidean-based methods significantly outperform their cosine-based counterparts (Fig. 3a). There is only a slight benefit, less than 0.05 on average, in *AUC* from employing an ensemble-based method compared to a single-shot (Fig. 3b). *MultiLOO-ISN*, *LOO-ISN* and *SSN-m* have similar performance for single-edge settings (Fig. 3c). Cook’s distance approaches are among the best performers when module size *k* is high; they are suboptimal for small module sizes (Fig. 3d,e).

#### Result by number of outliers *M*

As shown in Fig. S3, the behaviour of *AUC* with respect to module’s size *k* is not affected by the number of outliers *M*. The relative ranking of methods appears to be consistent with respect to the number of outliers *M*, with range *M* = $\{1,5,10 \}$. Increasing the number of outliers *M* worsens the performance of all methods: no method shows high robustness to outliers. Finally, the performance heterogeneity, i.e., the spread between best and worst methods, increases slightly when the number of outliers *M* increases.

### Results on real-life data: the LucKi Gut subcohort

Microbiome co-occurrence networks are known to be rich in terms of information on the health conditions of individuals^4,33^. Hence, we use data from the LucKi Gut cohort, an ongoing study that monitors gut microbiota development throughout infancy and early childhood, to validate the findings.

The LucKi Gut is embedded within the larger Lucki Birth Cohort Study^28^; it mainly focuses on newborns, collecting microbial taxa at various stages after delivery and thus computing microbial associations. Microbiome at month 6 has been identified as a milestone in microbial community maturation; hence it constitutes the subject of the analysis. We focus on the 81 newborns having microbial profiles available at month 6 and, through significance assessment methods, we try to discover which are the outlying individual-specific modules if any. We apply filtering based on the prevalence of microbial taxa ($< 10\%$). All the samples have substantial sequencing depth (reads: median = 57,248, IQR = 29,504; minimum = 11,123); hence we do not apply any filter on the number of reads. The resulting data are composed of 81 newborns per 126 microbes. We centered-log ratio (CLR) transformed the data and computed the Pearson correlation network on the whole dataset, i.e., the population-based network.

The considered modules are the clusters obtained by applying the community detection algorithm *SPINGLASS*^29^ on the population-based network. We do not apply any binarization or distribution-based transformations. We set the parameter *stop temperature* to 0.001 to increase the algorithm’s granularity, while the other parameters are set to their default values.

We apply *SPINGLASS*^29^ to the Lucki Gut cohort, and we find 4 microbiotic modules of dimension {45, 41, 35, 5} taxa. Modules 1–3 have a size (number of taxa) out of the coverage of the simulations ($>17$). Moreover, modules 1–3 consist of more edges, as computed according to^27^, than individuals 81, and thus all methods based on Cook’s distance can not be used. Module 4, consisting of 5 nodes, is adequate to validate our approach, being the closest to the module’s dimensions in the simulations.

**Figure 4:**
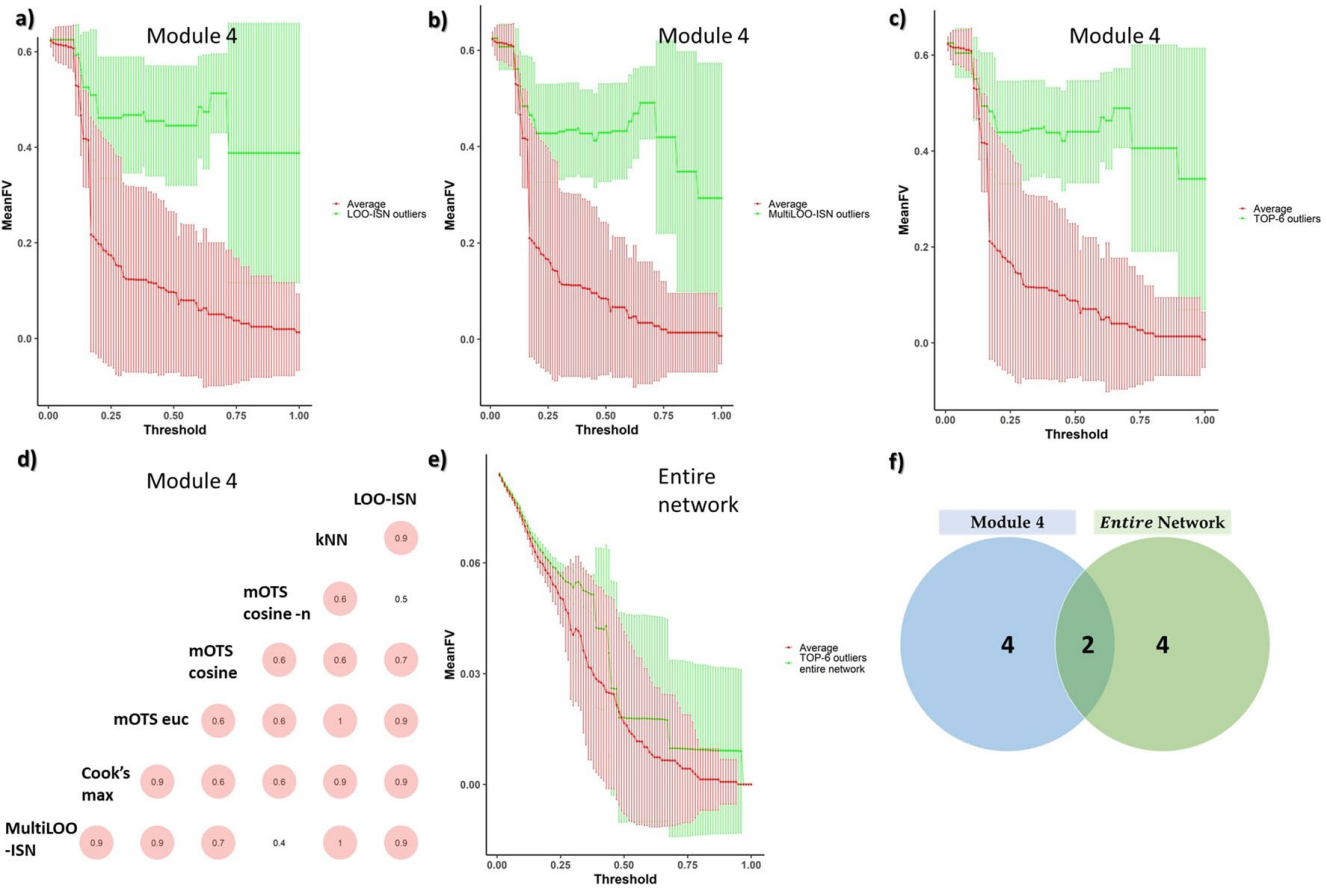
Real-life data: the LucKi Gut Cohort. Filtration curve of *ISNs* using *Fiedler values* as the metric. The standard deviation is also depicted. In module 4, (**a**) the filtration curves of 4 outliers (green) identified with *LOO-ISN* are depicted against the rest (red, identified as ”average”). In (**b**), the green line is the filtration curve of 7 outliers identified with *MultiLOO-ISN*. In (**c**), the top-6 outliers, as for the geometric mean of the ranking, are depicted (green) against the rest (red). In (**d**), the agreement, in terms of correlation of *OS*, is calculated between the specified methods. In (**e**), the top-6 outliers on the entire network are depicted (green) against the rest (red). (**f**) Only 2 samples out of the top-6 in the entire network are also outlying in module 4.

Hence, we apply outstanding techniques from synthetic data on the *ISNs-L* of module 4. In particular, *kNN 5* $\sqrt{N}$, *mOTS euc*, *MultiLOO-ISN*, *LOO-ISN*, *Cook’s max* *mOTS cosine*, and *mOTS cosine -n*. *MultiLOO-ISN* and *LOO-ISN* find 7 and 4 significant outliers respectively, visualized in Fig. 4a,b. We create an ensemble ranking of the individuals through rank comparison. A comparative study from Li et al.^34^ guides us toward the geometric mean of the rankings, among the best metrics in terms of performance and generalizability. There is strong agreement between the *outlier scores* of different methods, with correlation in absolute value higher than 0.4 (Fig. 4d). We focus on the top-6 as for the geometric mean (Fig. 4c). We choose 6 as it is between 7 and 4 outliers found with *MultiLOO-ISN* and *LOO-ISN*.

Further validation comes from *graph filtration curves*^35^, i.e., graph representations that can be applied to labelled and unlabelled datasets using the graph’s relevant attributes and structural information. An increasing threshold is considered, and those *edges* whose weight is smaller than the current threshold value are zeroed out. A metric summarizing the subgraph is then calculated for different threshold values. In more detail, we use the algebraic connectivity of graphs, the so-called *Fiedler value*^36^; it measures how well a graph is connected^37^. Further information can be found in the Supplementary.

In Fig. 4a–c we depict the outlier groups against the average of all other *ISNs-L* in the population, thus showing strong separation in the given module. Hence, corroborating *LOO-ISN*, *MultiLOO-ISN*, and top-6 outliers findings.

We compare the top-6 outliers with external phenotypes such as the mode of delivery (Vaginal or C-Section) or the diet type (Breastfeeding, Mixed diet, Solid food). We find enrichment for Solid food diet and mildly for C-section delivery (hypergeometric tests, respectively, p-value of 0.032 and 0.079, with FDR correction).

Then, we consider, as an extreme, the entire network as a module (Fig. 4e). We iterate the pipeline on the entire network (i.e., on the ISNs of 81 individuals and 126 taxa) and rank the top-6 outliers (as before). We note that the *Cook’s max* was not calculated since there are more edges than samples. We find significant enrichment for Solid food in the top-6 (6 out of 6, hypergeometric FDR corrected p-value of 0.032). No delivery type level is enriched. Moreover, 5 out of the top-6 samples are in Cluster 2 of the *DMM* cluster analysis of Gallazzo et al.^38^ on the same data. However, we find no enrichment (FDR-corrected hypergeometric p-value $= 0.35$). We do not find any separation in the filtration curves by taking Module 4’s top-6 outliers on the entire network. Out of Module 4’s top-6 outliers, only 2 are also outlying on the entire network (Fig. 4f).

## Discussion

Individual-specific networks have become increasingly popular. In general, an ISN refers to a network that can be allocated to a single individual. As such, a systems approach can be adopted to compare individuals with each other and to assess heterogeneity in patient or population groups, which may inform precision medicine practices. Here, we focus on ISNs with edges that have individual-specific weights. Often such ISNs also have individual-specific node values, as these are directly used in the computation of the edges. However, one can think of examples for which node values would not be directly available. For instance, individual-specific gene-level statistical epistasis networks could capture the individual’s epistatic contribution to a population epistasis model, in the sense of Kuijjer et al.^13^. However, individual-specific gene-node values would only be available when the derivation of the population epistasis model involves the computation of gene summaries. Once ISNs are derived, they can be interrogated for highly connected subnetworks. When ISNs are molecular, they can be followed up by enrichment analyses to identify individual-specific significant pathways. However, before embarking on such analyses, we first investigate whether the individual should be treated as a unique sample or whether the individual can be assumed to follow population trends. Thus conclusions from population models can be extrapolated to the individual without further ado. Currently, ISNs are often subjected to interpretation workflows, irrespective of whether or not edges or modules are significantly different from what can be expected from a population. Hence, this work explores several outlier detection methods, formulates new ones, and translates them into the context of ISNs, going beyond single-edge significance assessments.

ISNs, with individual-specific edges, can be computed in various ways. We have restricted attention to Kuijjer’s linear interpolation method as the construction method can be applied to any definition of an edge. This does not imply that any edge weight definition will give optimal performance. Kuijjer reported^13^ (and Jahagirdan^39^ as well) that noisier results are obtained with mutual information edge weights. In addition, each application setting will require a thorough evaluation of the appropriateness of the adopted ISN definition on simulation data that capture the true nature of the target application data. When applied to Pearson’s correlation as a measure of association between two nodes, Kuijjer’s individual-specific edges are quite similar to those defined by Liu et al.^21^ (SSN-n). The latter did develop a Z score (*SSN-m*) from ISN edges to assess significance. However, type I error for SSN-n was slightly elevated. For the reasoning behind this observation, we refer to Jahagirdan et al.^27^.

This paper presents several methods from different research fields to assess which individual is significantly different from the population, where the population is described via a network of interacting biological entities (for instance, genes and their expressions or microbes and their abundances). As entities often do not work in isolation, we have extended current state-of-the-art sample outlier detection methods to work conditional on interconnected sets of measurements for each individual. Hence, in the simulations, we do not look for modules but condition on a given subnetwork, then check if the individuals are outliers conditional on the subnetwork. Outlying individuals, not on the whole networks but on a subset, identified with our techniques may point towards interesting subnetworks of ISNs to zoom into for follow-up analyses. Realizing that nodes, being they genes, taxa, or any other biological features, do not act in isolation (but in communities), we expanded the current state-of-the-art analysis toward a modular significance paradigm.

In our work, we have clearly specified the null and alternative hypotheses we are testing with each considered outlier detection method. Our simulated data mimics two real-life scenarios: (1) transcriptome (gene co-expression) networks for normal distribution and (2) microbial co-occurrence networks for compositional distribution. The motivation to select these two application contexts is as follows. Gene co-expression is the field in which ISNs have mainly been applied. Moreover, as highlighted by Conesa et al.,^40^, the read counts are best modelled with discrete distribution (as the Poisson or negative binomial^41,42^). However, as soon as the data have been normalized—including TMM and batch removal—they might lose their discrete nature and be more akin to a continuous distribution. Moreover, there are numerous advantages that the Gaussian distribution provides, such as being a natural representation of an average for large sample sizes, to completely independent mean and deviance. The normality assumption could be an issue, thus its use is limited only to scenarios where the assumption holds. For those particular fields, a custom data generation technique is needed. The microbiome has a considerable impact on health^43^. Furthermore, the human gut is a complex ecosystem where microbes interact amongst themselves, and with the host^33^. Microbial interactions have been shown to exhibit rich information about various health conditions potentially^33^.

In the era of data science and precision medicine, robust outlier detection is of great interest^44,45^. Determining whether an observation is unlikely, given the available data or a reference, clearly is context-dependent. In our context of ISNs, which are networks, it makes more sense to look for outliers in a multivariate way, where a multivariate outlier is classically defined as an observation that is inconsistent with a given correlation structure. The complexity of multivariate outlier detection is exacerbated in the context of ISNs, which may consist of thousands of edges. To reduce the complexity and, since modules are often the basic units towards interpretation and translation, we restrict the dimensionality of multivariate outlier detection to those dictated by modules. Hence, we focus on low-dimensional simulations, to replicate the dimensionality of a real-life module. Our selected outlier detection methods are representatives from *kNN*, *OPTICS*, *Spoutlier*, *Cook’s distance* and *SSN-m* families, and are unsupervised: *kNN* and *Spoutlier* have different assumptions but are both distance-based techniques, while OPTICS is density-based. *SSN-m* (as *LOO-ISN* and *MultiLOO-ISN*) is based on leave-one-out, while *Cook’s distance* is both statistical and distance-based. Methods initially developed for univariate (multivariate) outlier detection are respectively *SSN-m* (*kNN*, *OPTICS*, *Spoutlier*, *Cook’s distance*). Although there is no best overall performer across scenarios, a few observations can be made: the increased dimension of the module is associated with stronger performances. Moreover, we observed a slight performance improvement by increasing the sample size. Distributional distance—on the compositional data—between outliers and non-outliers is a critical performance driver. Particularly the parameters *Mult* and *Data heterogeneity*. Settings where *Mult=1.1* are extremely challenging for all methods, with the value of the *Median AUC* ranging from 0.5 to 0.51. Under this scenario, the discrepancy between *cases* and *controls* is feeble, and it is not detected by any method. Hence, it is crucial to further analyze the characteristics of the target dataset before applying outlier detection methods in a myopic manner.

We can formulate interpretations and insights based on the methods’ performance. We show that the proposed methods, i.e., the Cook’s distance methods applied on the edges, *Cook’s max* and *Cook’s med*, are the first choice. Cook’s methods are among the best with *mOTS* cosine under the synthetic data normality assumption setting, with *kNN* and *LOO-ISN* under the synthetic data compositional setting. On the contrary, *OPTICS* is consistently a poor performer. A possible explanation for such a result is that Cook’s construction can give the proper importance to the ecosystem view characterizing network medicine. Indeed, when computing the influence/extremeness of an edge, it considers the entire modular structure. Moreover, we can deduce that we can tackle edges in transcriptomics data from a geometrical point of view. This is clear from the better performance of *mOTS* cosine than the euclidean counterpart. The algebraic approach—*mOTS* euclidean—has better results on microbiome simulations.

Alternatives to proposed outlier detection methods exist. (Non-linear) dimensionality reduction methods such as (non-linear) PCA, (kernel-) MDS, or SNE, on cell entries of the upper diagonal association matrix linked to each ISN, can be used to identify outliers as well, albeit primarily by visual inspection only. Some clustering approaches are robust to outliers in the sense that they will identify outliers as a separate cluster: One recent development that is promising in the context of ISNs is netANOVA, a novel hierarchical network clustering approach with tree-based significance assessment^46^.

Real-life data confirms our findings. The study on the LucKi Gut cohort microbiota data validated the proposed outlier detection methods in finding local outliers, i.e., observations that are not global outliers but become outliers only when they belong to specific feature communities. This is crucial in microbiomes, given their substantial heterogeneous structure and the importance of their variation^47^. Moreover, by doing an ensemble of the most performant techniques on the smallest module (i.e., module 4), we can segregate the diet type and the mode of delivery. In particular, the C-sections mode of delivery is known to be a prime driver for microbiota in the early stages of life^48–51^. This highlights the capacity of capturing a signal of the aforementioned methods. Moreover, the top-6 most outlier individuals in module 4 are not outlying in the whole network Fig. 4e. Hence, local outlier detection brings complementary information.

Most of the presented methods are rankers, i.e., yield a ranking of the outlierness, while leave-one-out methods ($LOO-ISN$ and $MultiLOO-ISN$) are proper classifiers, i.e., provide a p-value. Even though p-values make it easier to pinpoint an exceptional sample, some of our best performers, i.e., *kNN*, *Cook’s distance*, and *Spoutlier*, did not provide such p-values. For rankers, more work is needed to translate a ranked list into decisions about which individuals are actually outliers. The computation burden varies across methods. MultiLOO-ISN and LOO-ISN are the most computationally intense single-shot techniques. OTS euclidean approaches are much slower than the OPTICS counterpart, highlighting the need for further optimization. Cook’s distance methods are fast, but their burden increases quickly with increasing module size. The full comparison on a module of size $k=5$ and with $N = 1000$ samples is shown in the Supplementary.

The selection of the reference data has been a point of discussion in the original papers introducing ISNs. For instance, in Kuijjer^13^, they investigated taking subsets of an initial reference set as background and showed that this had little impact on an individual’s specific network constructed from this background, especially when sample sizes increased (Kuijjer et al.,^13^). Similarly, Liu et al.^21^ also evaluated the impact of changing reference sets, concluding that the method is robust to smaller reference sets. In Jaha et al.^27^, they evaluated different reference set choices. Particularly the impact of doing a case-only, control-only, or pooled reference set. They concluded that using control-only reference sets in prediction is advantageous, but reduces the ability to generalize. However, in this work, the choice of reference data was straightforward. It is impossible to use case- or control-only reference sets in unsupervised settings. There might be problems arising from the variability of the reference set. If the samples of the reference set are a mixture of different populations, the results would be impacted. The impact of the choice of reference data on outlier status or downstream analysis of significant ISNs is the subject of future work. A follow-up project aims to find homogeneous reference sets as groups of samples sharing the same association pattern.

Finally, once interesting individuals have been singled out, these can be analyzed in a precision medicine context to identify biomarkers or provide mechanistic insights. Consistently with Jahagirdan^39^, we observe that class accuracy is already very high when using the edge values (unpublished). We conjecture that it is beneficial to go the furthest from an average edge representation (i.e., Pearson correlation). In this work, we go beyond the straightforward use of edge values as predictors, applying more sophisticated methods. More advanced methods can also be employed, such as graph representation learning.

In conclusion, ISNs are promising constructs. Their uptake in precision medicine contexts will rely on advancements to interpret ISNs, but also assessments to identify outlying or exceptional individuals. Such individuals could benefit from diagnostics or interventions based on their ISNs rather than on generic population models. This work shows the added value of module-based outlier detection methods over commonly used single-edge approaches.

## Methods

### Real data

#### Microbiome data: LucKi Gut subcohort

To validate the proposed methods, we used data from the LucKi Gut cohort, an ongoing study that monitors gut microbiota development throughout infancy and early childhood. LucKi Gut is embedded within the larger Lucki Birth Cohort Study^28^. Metagenomic DNA was extracted with a custom protocol involving mechanical and enzymatic lysis^52^. The primary analysis step on the samples was microbial profiling by next-generation sequencing of the 16S rRNA V3–V4 hypervariable gene region. Then, a DADA2-based pipeline was used to identify Amplicon Sequence Variants. The result of those steps is a collection of 1144 taxa abundances. Mainly, we focused on microbial associations on newborns collected at month 6 after delivery, identified as a milestone in microbial community maturation, further restricting attention to the 81 newborns with microbial profiling available.

Selecting informative individuals and taxa and filtering out random noise was achieved with an abundance and prevalence filter. Only amplicon sequence variants with a prevalence exceeding 10% survived the filtering. Filtering has been recognized as a crucial step in microbiome^53^, and we selected 10% in accordance with^53^. Only 126 (out of 1144) taxa remained. On the prefiltered data, we applied centered-log-ratio (*CLR*) transformation.

### Construction of individual-specific networks

In general, a network can be represented by a graph $G=(V,E)$ where *V* denotes a finite, non-empty set of *p* nodes and *E* is a subset of $V\times V$ containing pairs of connected nodes $e_{ij}:=(v_i,v_j)$ referred to as edges. In weighted networks, each edge $e_{ij}$ is associated with a weight $w_{ij} \in R$. See also Supplementary Table S1. For individual-specific networks, we assume that for each individual *q* $(q=1,\ldots , N)$ a unique network $G_q=(V_q,E_q)$ exists, where *N* is the number of individuals within the study cohort. Additionally, a subnetwork/module $G^\prime =\left( V^\prime ,E^\prime \right)$ is a network such that $V^\prime \subseteq V$ and $E^\prime \subseteq E$.

The individual-specific networks considered in the study were derived via Kuijjer’s LIONESS^13^ (see also Fig. S4), giving rise to undirected, weighted, individual-specific networks for each individual in the study, with strong properties, performances and adaptability in different contexts^13,27,54,55^. Hence, in our work, an individual-specific edge weight $w_{ij}^q$ for the individual *q* is computed with the following formula:

1 $$\begin{aligned} w_{ij}^q=N\ w_{ij}^\alpha - (N-1)\ w_{ij}^{\alpha -q} \end{aligned}$$

where $w_{ij}^\alpha$ is the edge weight in the population-based network and $w_{ij}^{\alpha -q}$ is the edge weight in the network calculated with the same measure of association (Pearson correlation in this work) but without the *q*-th observation i.e., the *LOO* network.

This formula exploits the difference between two networks, in which the only variation is the absence-presence of individual *q*, to draw conclusions about the impact on network topology of removing or adding an individual. Furthermore, the inspiration for the formula lies in the desire to construct ISNs such that their average would be close to the network constructed by pooling all study individuals together. The original paper effectively demonstrates that, with ${N\rightarrow \infty }$ and under the assumption that the ratio of weights is constant between population-based and *LOO* networks, linearity holds, and the population-based network can be seen as a weighted average of the *ISNs* (see^13^, Suppl. 5.2).

The *SSN-n* network is defined by the core difference $w_{ij}^\alpha -w_{ij}^{\alpha -q}$. The original paper^21^ based the reference set on the control samples, but it has been further extended in^27^ on the entire population. Since we are in an unsupervised setting, we used the latter definition.

### Hypothesis and outlier detection methods

*SSN-m*, *LOO-ISN*, and *MultiLOO-ISN* yield a p-value, while *OPTICS*, *kNN*, *Spoutlier* are rankers, i.e., yield an outlier score. The characteristics of the methods are highlighted in Table 4.

It is essential to clarify the underlying null hypothesis to find the *outliers*—individuals that deviate from the population-based association structure. Specifically, for a given edge $e_{ij}$:

2 $$\begin{aligned} H_0:E(w_{ij}^q)=\ E(w_{ij}^{\alpha })\ \end{aligned}$$

This formulation shows the direct link between $w_{ij}^q$ and $w_{ij}^{\alpha }$. If $H_0$ is not rejected, then the *population-based* conclusions are directly applicable to the *q*-*th* individual. If the test falls into the two-tails rejection zone, the individual is considered to be an *outlier* for the target edge/module. The above formulation Eq. (2) is directly generalizable to a module by extending the equality for every edge inside a module. We take *Md* as a module and define $Me = \{w_{ij} :i,j \in Md\}$ as the set of edge weights belonging to a module. Hence, the null hypothesis is:

3 $$\begin{aligned} H_0: \forall (i,j) \in Me, E(w_{ij}^q)=\ E(w_{ij}^{\alpha })\ \end{aligned}$$

Any strong deviation from Eq. (2) (Eq. 3 in modular assessments) is part of $H_A$. Depending on the method, the formulation of $H_0$ varies: (1) for *SSN-m*, $H_0$ refers to the equality of edges calculated on the reference network and a network with the addition of the sample *q*. In the subsection on *SSN-m*, we show the equivalence of this test with Eq. (2). (2) For *LOO-ISN* and *MultiLOO-ISN*, the null hypothesis is Eq. (2) (Eq. (3) if we test module significance). Further details are in the *LOO-ISN* and *MultiLOO-ISN* subsections. The other methods (3), *kNN*, *OPTICS*, *Spoutlier*, *Cook’s distance*, do not follow a classical hypothesis testing setting, i.e., they do not yield p-values or statistical significance. They assign a score, the *outlier score*, for each individual’s edge/module. The ranking of the *outlier score* provides a quantification of the degree to which an individual’s edge/module is outlying.

If $H_0$ is not rejected, no claim can be made on the edge/module tested as outliers. Hence, the target edge/module does not need to be characterized individually, and the population-based aggregation is the best estimation. Notably, from Eq. (1), we find that Eq. (2), is a necessary and sufficient condition for:

4 $$\begin{aligned} H_0:E(w_{ij}^q)=\ E(w_{ij}^{\alpha }) \Leftrightarrow E(w_{ij}^{\alpha }) = E(w_{ij}^{\alpha -q}) \end{aligned}$$

**Figure 5:**
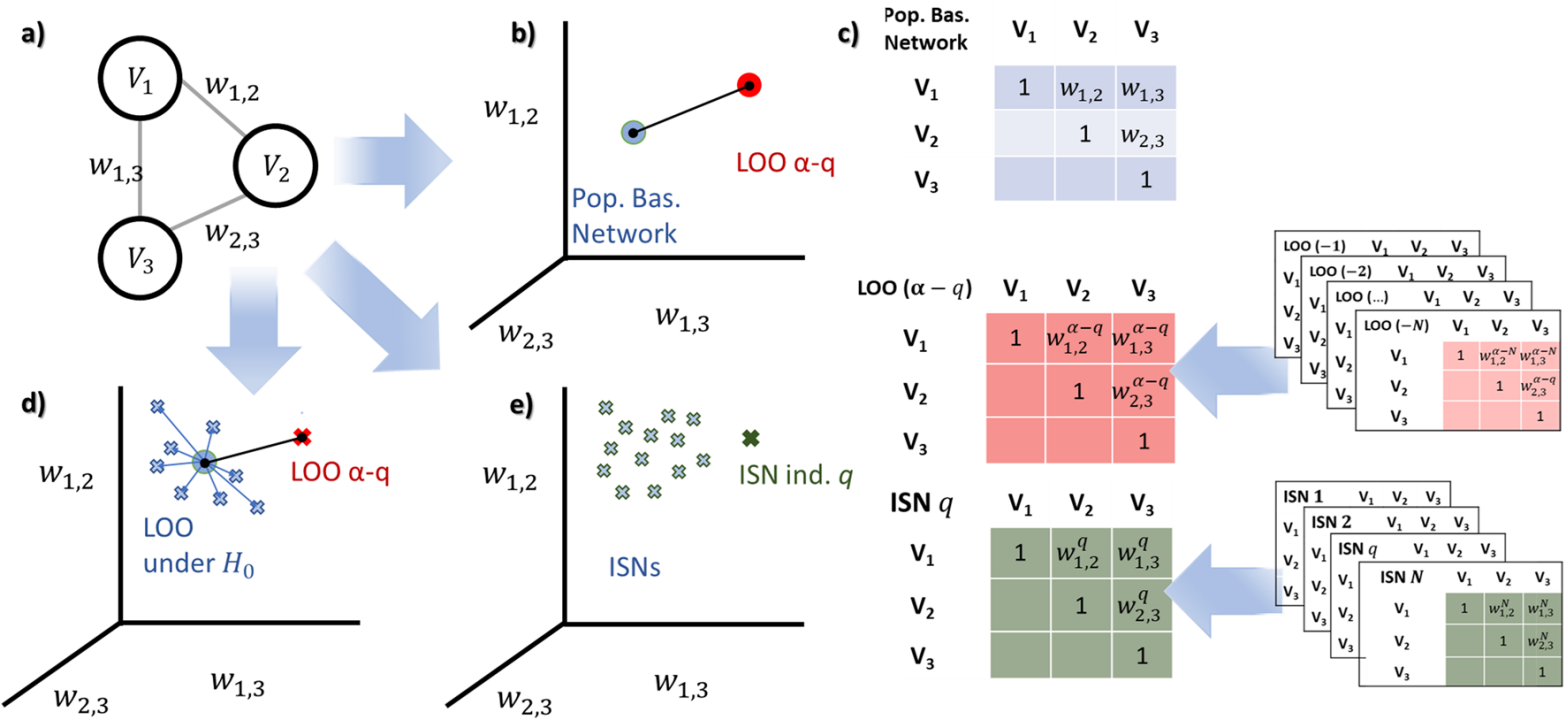
Three different testing scenarios to assess whether an individual is extreme compared to a population that is represented by a fully connected weighted network (i.e., all nodes are connected). (**a**) The example shows a network of 3 nodes (3 edges). This could be a module as a subnetwork of the larger population-based global network. In (**b**), the distance between the *LOO* network and the population-based network is computed. Note that when the population-based network is inferred from *N* individuals, the *LOO* network is based on $N-1$ individuals. SSN-m uses this testing scenario, but is limited to the bivariate case (2 nodes and 1 connecting edge). In (**c**) we can see the three types of networks considered: population-based, LOO, and ISN, highlighted for individual *q*. Similarly to (**b**), in (**d**) the distance between the *LOO* network and the population-based network is confronted with, this time, the distance between the population-based network and the expected *LOO* under the null hypothesis of Eq. (4) $E(w_{ij}^{\alpha }) = E(w_{ij}^{\alpha -q}$). Null networks are generated by sampling features set for *N* individuals from the estimated variance/covariance matrix, hence with no difference between the individuals, that provokes no difference in the network’s edge weights. This testing scenario applies to the MultiLOO-ISN and LOO-ISN implementations, where we provide further information about the null network sampling. Figure S5 shows the pipeline in detail. Lastly, for (**e**), the target individual’s specific network is compared to the ISNs of other individuals in the population, leading to an outlier score for the target individual. The network edge weights are used in kNN, Cook’s distance, OPTICS, and OTS outlier detection algorithms.

Hence, testing between population-based and individual-specific edge weights is equivalent to testing between population-based and *LOO* networks under the *ISNs-L* formula. A graphical overview of the significance assessment strategies can be found in Fig. 5.

**Table 4:** Main characteristics of employed methods to assess an ISN’s significance.

Method	Edge-assessment	Module-assessment	Parameters to tune	Assumptions	p-value	Fast description
LOO-ISN	TRUE	TRUE	*Rep*: number of repetition	Multivariate edge normality	TRUE	A null distribution is computed based on bootstrap resampling assuming Normality—*LOO* procedure— Aggregation method: sum of the absolute difference
MultiLOO-ISN	TRUE	TRUE	*Rep*: number of repetition	Multivariate edge normality	TRUE	As above—only difference the aggregation is non-linear: maximum deviation from the null in all edges
kNN	TRUE	TRUE	$k_{min}$, $k_{max}$ neighbour	/	FALSE	The distances found in kNN with k = $k_{min}$ to k = $k_{max}$ are averaged to create outlier scores
Optics	TRUE	TRUE	*MinPts*: number of neighbour	/	FALSE	Outlier score is computed via a radius distance of core and board points
Spoutlier	TRUE	TRUE	s: number of references	/	FALSE	The outlier score is calculated as the minimum within a small set of references observations kNN based
SSN-m	TRUE	FALSE	/	Edge normality	TRUE	The p-value is calculated as a transformation of the difference between $w^\alpha$ and $w^{\alpha -q}$
Cook distance	FALSE	TRUE	/	LM assumptions	FALSE	The outlier value is calculated aggregating cook’s distance in every individual trying to predict one of the edge weights in the modulus.

#### SSN-m

SSN-m^21^ calculates a p-value as a transformation of the difference between $w^{\alpha }$ and $w^{\alpha +q}$. $w^{\alpha +q}$ is an edge weight calculated by adding an individual before computing the correlation. *SSN-m* has been developed in a differential network paradigm and bounded to it. The network was calculated by *adding* a *q* observation, not *removing* it as in LIONESS. This discrepancy is not a problem, as the two situations (adding or removing an observation) can be reconciled by changing the point of view. Defining $PCC_n$ as the *Pearson* correlation of two nodes calculated on *n* observations, we define $\Delta PCC_n = PCC_{n+1}-PCC_{n}$ as the difference in correlation when adding observation *n+1*. It is straightforward to reconcile with the LIONESS situation, setting *(n+1) = N*, and then removing one observation yielding *n = N-1*. The p-value computation is based on a *z-score*, calculated as:

5 $$\begin{aligned} Z=\dfrac{\Delta PCC_n}{(1-PCC_n^2)/(n-1)} \end{aligned}$$

The underlying assumption is the normality of the distribution.

#### LOO-ISN

*LOO-ISN* belongs to the leave-one-out family. In the single-edge ($k=2$) setting, with nodes $v_i$ and $v_j$, and under the null hypothesis $H_0$ given by Eq. (2), the *LOO-ISN* method performs the following steps; (1) Use the *dataset of analysis* ($N \times k$ matrix containing node values) to compute the population-based network, with single element $w_{ij}^{\alpha }$, i.e., with Pearson correlation in our work; (2) Generate *simulated data*, i.e., *N* observations from a bivariate normal distribution with zero mean ($\mu = 0$), unit variance, and correlation equal to $w_{ij}^{\alpha }$; (3) Use *simulated data* to compute $\hat{w}_{ij}^{\alpha }$; (4) Remove one sample (*ind*) from the *simulation data* and compute the correlation $\hat{e}_{ij}^{\alpha - ind}$ on the remaining data; (5) Compute the difference between $\hat{w}_{ij}^{\alpha }$ and $\hat{w}_{ij}^{\alpha - ind}$; 6) Remove the individual *q* from the dataset of analysis and compute $w_{ij}^{\alpha -q}$, for each $q=1, \cdots , N$; 7) Compare $w_{ij}^{\alpha } - {w_{ij}^{\alpha - q}}$, computed on the dataset of analysis, to $\hat{w}_{ij}^{\alpha } - \hat{w}_{ij}^{\alpha - ind}$, computed on *simulation data*, for obtaining an associated p-value. As previously mentioned in Eq. (4), it is equivalent to testing between population-based and individual edges or between population-based and *LOO* edges.

The above steps, describing the pipeline for the significance assessment of a single edge ($k=2$), straightforwardly generalize in the case where a module ($k>2$) is considered. However, in such a case, we use a multivariate normal distribution for generating the simulation data in step 2), where the dimension of the normal distribution equals the module’s size *k*. Multivariate normal simulations need to mimic the network’s structure under the null hypothesis $H_0$. Hence, we generate *N* samples, equal to the empirical sample size, with a normal where we set the variance/covariance matrix to the adjacency matrix *A*, with entries the weighted edge weights $w_{ij}^{\alpha }$ and the mean vector ($k \times 1$) to 0. Hence, the correlation coefficients are estimated on the dataset of analysis, i.e., the edge weights $w_{ij}^\alpha$ for every edge between two nodes $v_i$ and $v_j$ inside the module. We refer the reader to Fig. S5 for a visual representation.

While the p-value calculation in step (7) is straightforward in a single-edge setting, multiple options are possible in the modular setting. *LOO-ISN* sums the differences across dimensions to create a *univariate* distribution and rejection zone. Hence, it tests the entire module.

We take *Md* as a module and define $Me = \{w_{ij}^q:i,j \in Md\}$ as the set of edge weights belonging to a module. For each individual *q* we defined the test statistic $T_q$ as:

6 $$\begin{aligned} T_q\ =\ \sum _{(i,j)\in Me}{(w_{ij}^\alpha -w_{ij}^{\alpha -q})} \end{aligned}$$

$T_q$ is then compared to the empirical distribution of the difference’s sum under the null hypothesis $H_0$, i.e., $\hat{T}=\sum _{(i,j)\in Me}{(\hat{w}_{ij}^\alpha -\hat{w}_{ij}^{\alpha -q})}$, and a p-value is obtained. For both LOO-ISN and MultiLOO-ISN the pseudocode explaining the various steps in detail is available in the Supplementary.

#### MultiLOO-ISN

*MultiLOO-ISN* follows the leave-one-out pipeline previously described, differing only on features’ aggregation. *MultiLOO-ISN* considers the module a point in a high-dimensional space and constructs a multi-dimensional rejection zone with as many dimensions as edges in the module. But, to calculate the test statistic, we need to reduce the rejection zone to a scalar. Hence, we apply *maximum* discrepancy (non-linear) to create a univariate distribution under $H_0$.

With *Me* the set of edge weights belonging to a module, for each individual *q*, we define the test statistic $T_q$ as:

7 $$\begin{aligned} T_q\ =\ \max _{(i,j)\in Md}{(w_{ij}^\alpha -\ w_{ij}^{\alpha -q})} \end{aligned}$$

We then compare $T_q$ to the empirical distribution of the difference’s max under $H_0$, i.e., $\hat{T}=\max _{(i,j)\in Md}{(\hat{w}_{ij}^\alpha -\ \hat{w}_{ij}^{\alpha -ind})}$ and retrieve a p-value.

*SSN-m*, *MultiLOO-ISN*, and *LOO-ISN* are strongly related: all assume normality, and take into account, as parameters, the sample size and the empirical population-based correlation $w_{ij}^\alpha$. They also show similar results on single-edge settings.

#### Spoutlier

*Spoutlier*^30^ is a fast implementation based on *kNN* logic. It defines a reference set and then calculates the distances between this set and the *q* target observation. Then it extracts the minimum of those distances, as the authors stated that an outlier is an observation far away from every observation in the dataset. The minimum of those distances is the *OS*. The only parameter is the number of individuals in the reference set *s*. We fix $s = 20$ accordingly to the suggestions in the original paper. In this work, we take the individual-specific edge weights in a module as our features.

Quantifying the *OS* for an observation part of the reference set of size *s* is a corner case. If no measures are taken, those individuals would have a distance of 0, not indicative of their outlier degree. In the original paper, the authors overcame this limitation by computing the lowest non-zero distance from the *s* reference set. This approach overlooks similarities in setting with high sample sizes and discrete features; In those settings, it is plausible to have multiple observations with the same profile, namely a *replicate*. If a *replicate* of the target observation is in the reference set, we should not discard a 0 distance. Hence, we introduced a minor modification of the original code. We sampled $s+1$ observations, and when the target is in the reference set, we use the other *s* observations. Otherwise, we randomly sample *s* out of $s+1$ observations.

The distance measure is crucial for the final performance. We propose cosine similarity (*OTS cosine*) to consider the geometrical nature of the data. *Dissimilarity* is computed through its complement. Cosine geometrical computation needs a multi-dimensional feature space and is unfeasible in single-edge settings. Considering the well-known paradigm of crowds’ wisdom^56^, we propose an ensemble technique. We iterate the algorithm (*mOTS cosine*, *mOTS euc*) multiple (10) times to have stabler performance. Moreover, we also propose a combination of euclidean and cosine similarity (*mOTS glob*). Hence, arithmetical and geometrical facets are combined. We implemented every method on both *SSN-n* and *ISNs-L* networks.

#### Cook’s distance

*Cook’s distance* is based on the module rationale. A module is a collection of strongly associated variables (possibly genes/taxa). Hence, *Cook’s distance* exploits shared information between the module’s components, being edges or nodes. The proposed adaptation of *Cook’s distance* predicts an edge weight via a linear model using all the other edge weights in the module as predictors for each iteration. Given a module of size *k*, with $k=$ number of nodes, the number of pairwise combinations (order not considered) between the edges is $C = \frac{k(k-1)}{2}$. In particular, for $q= \{1,\ldots ,N\}$ and $c=\{1,\ldots ,C\}$, we use a linear model (LM) to predict an edge weight $w_{ij}^q$ with every other edge weight $w_{lm}^q$ in the module, $(l,m) \in \{1, 2, \dots , k \}^2$ with $(l,m) \ne (i,j)$ and $l < m$:

8 $$\begin{aligned} w_{ij}^q = \beta _0 + \Sigma _{(l < m)}^{C}\beta _{l m} w_{l m}^q + \epsilon _q \end{aligned}$$

Then, we apply Cook’s distance to identify which observation is outlying (high residual) and with substantial leverage, namely having a strong influence on the estimation of $w_{ij}^q$. Cook’s distance for an observation *q* and edge weight $w_{ij}$ (connecting nodes $v_i$ and $v_j$) as the target, is defined as follows:

9 $$\begin{aligned} D_{ij}^q=\ \frac{\sum _{p=1}^{N}{({\hat{w}}_{ij}^p-\ {\hat{w}}_{ij}^{p(q)})^2}}{(C+1){\hat{\sigma }}^2} \end{aligned}$$

where $\hat{w}_{ij}^{p(q)}$ is the fitted response value obtained when excluding individual *q*, with

10 $$\begin{aligned} \hat{w}_{ij}^q = \hat{\beta _0} + \Sigma _{(l < m )}^{C}\hat{\beta }_{l m} w_{l m}^q. \end{aligned}$$

The computation of Cook’s distance is iterated for (1) each observation, yielding $D_{ij}^1$, $\ldots$, $D_{ij}^N$, and 2) for each edge in the module as a target, yielding $D_{12}^q$, $\ldots$, $D_{k-1k}^q$. Finally, for each observation *q*, we aggregate all the $D^q$={$D_{ij}^q$ with $j=2,\ldots , k$, $i=1,\ldots , k-1, i<j$} to find the global *OS*. Mean, median and maximum-based aggregation on the iterations are proposed.

#### kNN

We focus on the implementation from Angiulli^31^. This extension has been developed for outlier detection. As features, we use all the edge weights inside a module *Me*. For further details, we refer to the original paper and the method section of the Supplementary.

#### DBSCAN: OPTICS

*OPTICS-OF* (simply referred to as *OPTICS* in the paper) is an enhancement of *DBSCAN* developed for outlier detection. The edge weights inside of a target module are the features. *OPTICS* yields an outlier score. For further details, we refer to the original paper and the Supplementary method section.

### Synthetic data

We used synthetic data to evaluate and compare the above methods where the ground truth is available. We created several heterogeneous settings with varying assumptions, generation schemes, and parameters. Notably, we employed two different generation schemes: (i) normal distribution and (ii) compositional scheme. In both schemes, we simulate the dataset of analysis (individuals on the rows, features on the columns, dimension $N\times k$) via different distributions’ parameters for the $N-M$ *controls* and the *M* *cases*, i.e., the outliers.

Shared parameters in both schemes are (1) sample size *N*, varying between 100 and 2000; (2) number of outlier individuals *M*, varying between 1 and 10 (in percentage from $0.05\%$ to $10\%$); (3) the module’s size *k* quantifying the number of nodes in the module, varying from 2, a single-edge scenario, to 17. In both normally distributed and compositional simulation schemes, we define a multivariate random variable [multivariate normal for (i)], and we sample each individual’s variable set (i.e., the nodes) from this multivariate distribution. Individuals are sampled independently, and all the control individuals are sampled from a distribution with the same parameters. Then, Pearson correlation is applied to the sampled dataset of analysis, to build the population-based network (single entry $w_{ij}^{\alpha }$). This population-based network is the input for the ISN computation. Moreover, we employed two different outlier generation distributions for the normal distribution scheme, specifying if the outliers belong to the same distribution or if each one comes from its’ own distribution. Microbial scheme’s specific parameters control: (1) the degree of data heterogeneity (varying from *uniform* to *high*); (2) the multiplying factor between *differing* microbes (from 1.1 to 2); and (3) the percentage of inflated parameters differentiating *cases* and *controls* (from $10\%$ to $40\%$).

We explored multiple parameter settings, in particular, on the data distributional assumption. A combination of parameters is stored in a grid. For each entry (row) of the parameter’s grid created in the data simulation steps, we performed multiple runs (200 in normal distribution and 150 in compositional). Hence, the dataset of analysis (individuals on the rows, features/nodes on the columns) and the ground truth are generated. For each of those runs, we applied all the presented methods and each of them yields the vector *OS*, with dimension *N*. This vector contains the *M* *cases* and $N-M$ *controls* and represents the outlier scores for the individuals. The label *GT* of each individual refers to group membership: belonging to the *case* (outliers) or *control* group. For each individual $i=1,\cdots , N:$

11 $$\begin{aligned} GT_{i} = {\left\{ \begin{array}{ll} 1 &{}\text {if Outlier}\\ 0 &{}\text {Otherwise} \end{array}\right. } \end{aligned}$$

#### Synthetic data: normally distributed

The data composing the dataset of analysis are sampled through a multivariate normal. The mean vector is fixed at zero, while the variance-covariance structure differs between *M* *cases* and $N-M$ *controls*. The parameter *k*, the module size, controls the normal’s dimensionality. Sampled *cases* and *controls* observations are joined and constitute the dataset of analysis, i.e., mimicking the expression of genes in our population. Individuals’ ground truth *GT*, is used to evaluate the performance of the proposed methods. A visual pipeline is shown in Fig. S6.

Base parameters are *N*, *M*, *k*, *Outlier generation*, and we refer to Table 5 for details. We generated data by varying multiple parameters and then expanding them in a grid where each row is a unique combination of the base parameters and referred to as a setting. In total, we generated 168 different settings via the parameter combinations. Generation and evaluation steps, i.e., applying proposed methods to the data, were repeated *Rep =* 200 times to lower noise and ensure robust and reproducible results.

#### Synthetic data: compositional

We extended the work of Harrison^57^, proposing a Dirichlet-based model to simulate microbial data. Firstly, we sampled from: (1) A *Pareto* distribution with threshold = 1 and $\alpha$ = 0.7; or (2) A *Pareto* distribution with threshold = 1 and $\alpha$ = 4; or (3) A *Uniform* distribution with value = 1. The Pareto distribution describes data with few abundant features and many rare features. Every node has equal probability mass in the Uniform distribution. Hence, we generated the vector *D*, with single entry scalar $d_i$ with $i=1,\cdots ,p$. *D* is an intermediate result used as the concentration parameter ($\alpha$) in the Dirichlet sampling. Having a vector of $d_i$ tells us how much probability mass to assign to each node, each taxon.

Then, *cases* and *controls* observations are differentiated through a multiplier (*Mult)*, to produce ${E^1}$ from *D*, with single entry scalar $e^1_i$. The multiplier inflates nodes’ probability mass in *cases* and ranges in $Mult=\{1.1,\ 1.5,\ 2\}$. The percentage of nodes we inflate is given by the parameter: $PercIncrease=\{\ 10\%,\ 25\%,\ 40\%\}$. As in the normality simulation scheme, we combined parameters in a grid. After the case-control differentiation, the parameters, i.e., ${E^1}$ and *D*, are standardized to the same sum to avoid scale effects due to different densities:

12 $$\begin{aligned} {e}_i^*={e}^1_i\frac{\sum _{i=1}^{p}{d}_i}{\sum _{i=1}^{p}{e}^1_i} \end{aligned}$$

with $i=1,\ldots ,p$, hence forming vector $E*$.

We then multiplied $E*$ and *D* for $Int=3$, the intensity parameter, to accentuate the differentiation. Then, for each of the $N-M$ control individuals, *D* is used as the concentration parameter in a *Dirichlet sampling*. For an individual *q*, Dirichlet sampling results in $pr^q$, dimension $p \times 1$. Combining all the $pr^q$ for the $N-M$ individuals, we obtain the *pr* matrix, of dimension $(N-M) \times p$, with single entry $pr_i^q$ the probability of taxa *i* in individual *q*. We used $pr^q$ as downstream input-parameter of a *multinomial* sampling procedure, for individual *q*, with an additional parameter *number of reads*$=5000$. *Number of reads* specifies the total number of objects to divide into *p* boxes (the nodes) in the *multinomial* sampling with $prob=pr^q$ vector of probabilities. This step mimics a microbiome read on an individual with *number of reads* = 5000 and a vector of zero-inflated, compositional, and heterogeneous probabilities. The produced result, for individual *q*, is a vector of abundances under the control setting. An analogous procedure is applied to generate the *M* *cases* individual with parameter ${E^*}$ instead of *D*. We join the abundances for the $N-M$ controls and the *M* cases into the simulated dataset of analysis. The exhaustive pipeline can be found in Fig. S7.

To avoid biased perfect negative correlations, we sampled a network ten times bigger (in terms of the number of nodes) than the target’s module, $p=10\times k$. Then, we applied a centered-log-ratio (CLR) transformation^58^. Only at the very last step do we focus on the target module. We ensured that said procedure conserves at least one differentiation yielded by *Mult* in the *k*-dimensional module. Otherwise, there is no theoretical justification for differences between cases and controls.

Table 5 highlights the final grid of parameter values. In total, we generated 972 different settings via parameter combinations. Generation and evaluation steps are repeated $Rep=150$ times for each setting to lower noise and ensure robust and reproducible results. Compared with normality’s simulations, parameters *N* and *k* varies over a limited set. This limitation compensates for adding microbiome-specific parameters and keeps the computation burden under control.

**Table 5:** Details of the hyperparameters used in the normal and compositional simulations.

Normal distribution			Compositional		
Parameters	Values	Details	Parameters	Values	Details
N	100, 500, 1000, 2000	Controls + cases observations	N	100, 500, 1000	Controls + cases observations
M	1, 5, 10	Cases observations	M	1, 5, 10	Cases observations
k	2, 3, 5, 7, 9, 11, 17	Module’s size	k	2, 5, 11, 17	Module’s size
Outlier generation	Common, Specific	Common: all outliers share a common distribution Specific: each outlier has a different variance-covariance structure.	Data heterogeneity	Uniform, $\alpha$ = 4, $\alpha$ = 0.7	Degree of heterogeneity of the parameter to generate the data, going from no heterogeneity (Uniform) to high heterogeneity (Pareto with $\alpha$ = 0.7 ) passing through mild heterogeneity (Pareto with $\alpha$ = 4 )
			Mult	1.1, 1.5, 2	Multiplying factor applied to a percentage of observation to differentiate between cases and controls observations
			Percentage increase	10%, 25%, 40%	Percentage of inflated parameters on the total differentiating cases and controls

### Evaluation and parameter tuning

The result of a method on a run is an outlier score vector *OS*. This vector is ordered descendingly and compared with the ground truth vector *GT* (1 if outlier, 0 otherwise). While fixing a threshold and binarizing *OS* would help the evaluation task, there is no known threshold or p-value calculation for most of the considered methods. The natural way to evaluate our results is by varying the threshold and creating the corresponding *ROC* curve. We aggregated the performances, averaging all the runs (200 normally distributed, 150 for microbial) for each setting. We used the median as the aggregation metric, given the variability and skewness of the performances.

Since most of those families have parameters to tune or different aggregations can be used, the number of implementations is huge. For consistency, we applied every method, when possible, on both *SSN-n* and *ISNs-L* for every parameter’s choice. In kNN, we defined 2 different sets of parameters $k_{min}$ and $k_{max}$. 1) Firstly, $k_{min}$ and $k_{max}$ are respectively the minimum and maximum between *log*(*N*) and *k*, with *N* sample size and *k* module’s size. This parameters’ setting summarizes both the variables and the samples space; Then (2), $k_{min}$ and $k_{max}$ are the minimum and maximum between 5 (seen as a baseline parameter for kNN) and *sqrt*(*N*), also taken as a baseline in^30^. In *OPTICS-OF*, we set the parameter *n*, i.e., number of neighbors, as for *kNN*, as 5, $\sqrt{N}$ or $mean(log(n), k+1)$, to summarize both module and sample size. *Spoutlier*’s only parameter, the dimension of the reference set, is set as $s=20$ as empirically found in the original^30^ paper. We implemented (1) Euclidean distance and (2) cosine similarity as distance measures. We applied ensemble techniques to the *mOTS* methods, repeatedly choosing the 20 baseline samples and aggregating the different results with the median. Considered aggregations in Cook’s distances were (1) max, (2) average, or (3) median across all edges in a module. All the combinations and approaches are described in the Supplementary.

## Supplementary Information

Supplementary Information 1.

Supplementary Information 2.

## Data Availability

The dataset underlying this article is available upon request from the Euregional Microbiome Center (www.microbiomecenter.eu). Simulation data, code and graphs are publicly available in the GitHub repository at https://github.com/FedericoMelograna/Sign_ISN.

## Code availability

The code and the simulations are freely available on GitHub at https://github.com/FedericoMelograna/Sign_ISN. For further information on the analysis, software, and visualization, we refer to the software analysis and visualization section in the Supplementary methods.
