# Supplementary material for: Edge and modular significance assessment in individual-specific networks
Source: Sci Rep. 2023 May 15;13:7868. doi: 10.1038/s41598-023-34759-8 (PMC10185658; doi:10.1038/s41598-023-34759-8)
Supplement: Supplementary file 1 — Supplementary Information 1. [file 41598_2023_34759_MOESM1_ESM.docx]

Edge and modular significance assessment in Individual specific network

Supplementary Document I

Federico Melograna^1,*^, Zuqi Li^1^, Gianluca Galazzo^2^, Niels Van Best^3,4^, Monique
Mommers^4^, John Penders^2,5^, Fabio Stella^6+^, and Kristel Van Steen^1,7+^

1 BIO3 – Laboratory for Systems Medicine, Department of Human Genetics, KU Leuven, Leuven, Belgium;
2 School of Nutrition and Translational Research in Metabolism (NUTRIM), Department of Medical Microbiology Infectious Diseases and Infection Prevention, Maastricht University, Maastricht, The Netherlands;
3 Institute of Medical Microbiology, RWTH University Hospital Aachen, RWTH University, Aachen, Germany;
4 Department of Epidemiology, Care and Public Health Research Institute (CAPHRI), Maastricht University,
Maastricht, The Netherlands;
5 Care and Public Health Research Institute (CAPHRI), Department of Medical Microbiology Infectious Diseases and Infection Prevention, Maastricht University, Maastricht, The Netherlands;
6 Department of Informatics, Systems and Communication, University of Milan-Bicocca, 20126, Milan, Italy;
7 BIO3 – Laboratory for Systems Genetics, GIGA-R Medical Genomics, University of Liege, Liege, Belgium;

*federico.melograna@kuleuven.be
+Equal contribution

**FIGURES**

**Generating ISNs from dataset of analysis**

**
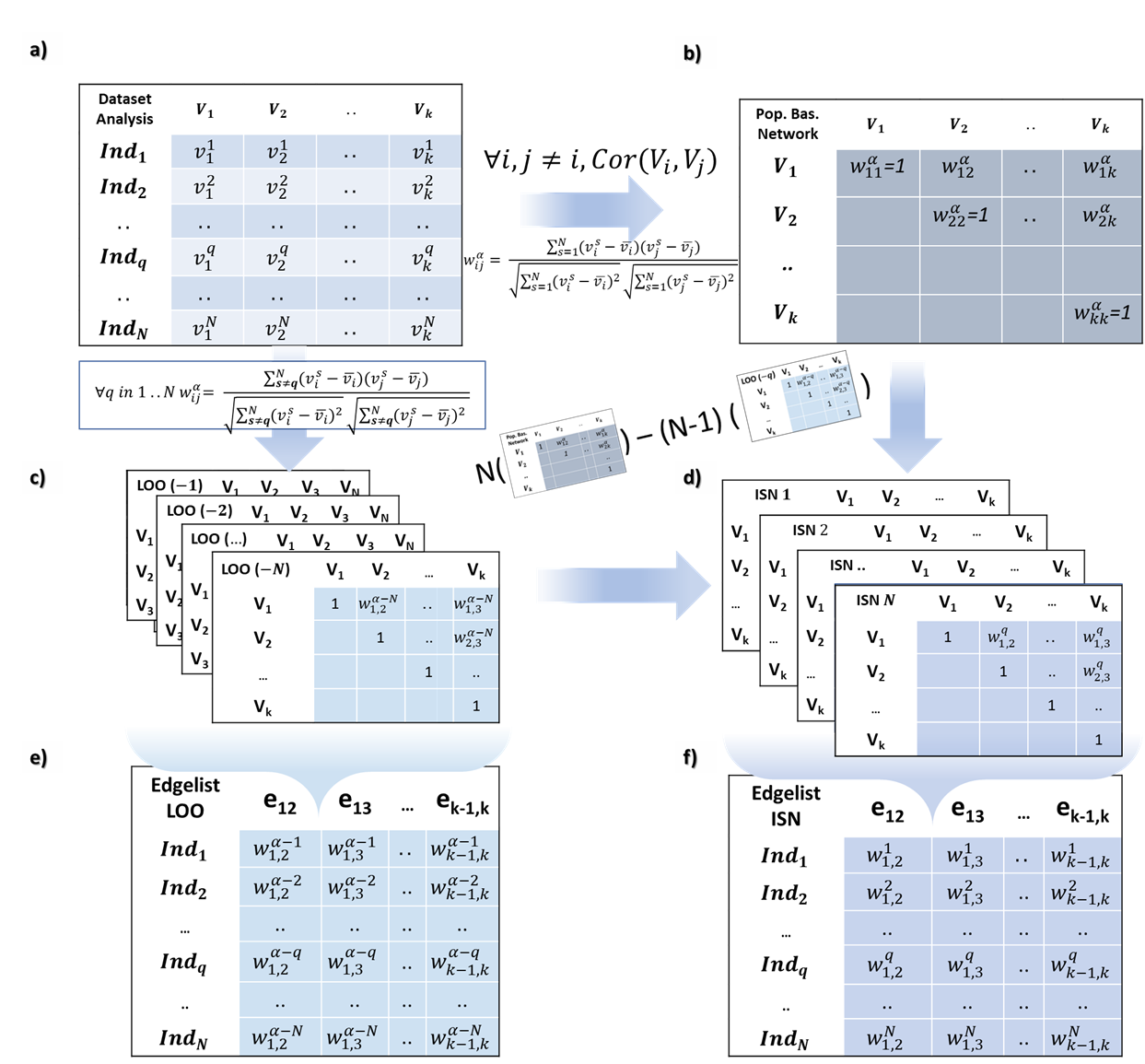
**

Figure **S1**: Steps from the dataset of analysis to the ISN and LOO feature sets. From the dataset of analysis, with $N$Individuals on the rows and $k$ features (nodes) on the columns, a) we compute the Pearson correlation, creating the Population-based network (b), with as a single entry the correlation between the features. Pearson correlation, but on all but one individual, is also performed c) to calculate the LOO networks. The ISNs are computed – here with the Kuijjer’s formula – confronting the population-based network and the LOO network, respectively multiplied by $N$ and $(N-1)$, with $N$ representing the sample size. Finally, the ISN and LOO edge weights are used as feature sets, and can be reorganized in a tabular format (e,f). Only the upper diagonal of ISN and LOO matrices is considered, since Pearson correlation gives a symmetrical matrix.

**Gaussian performances varying on k and M**


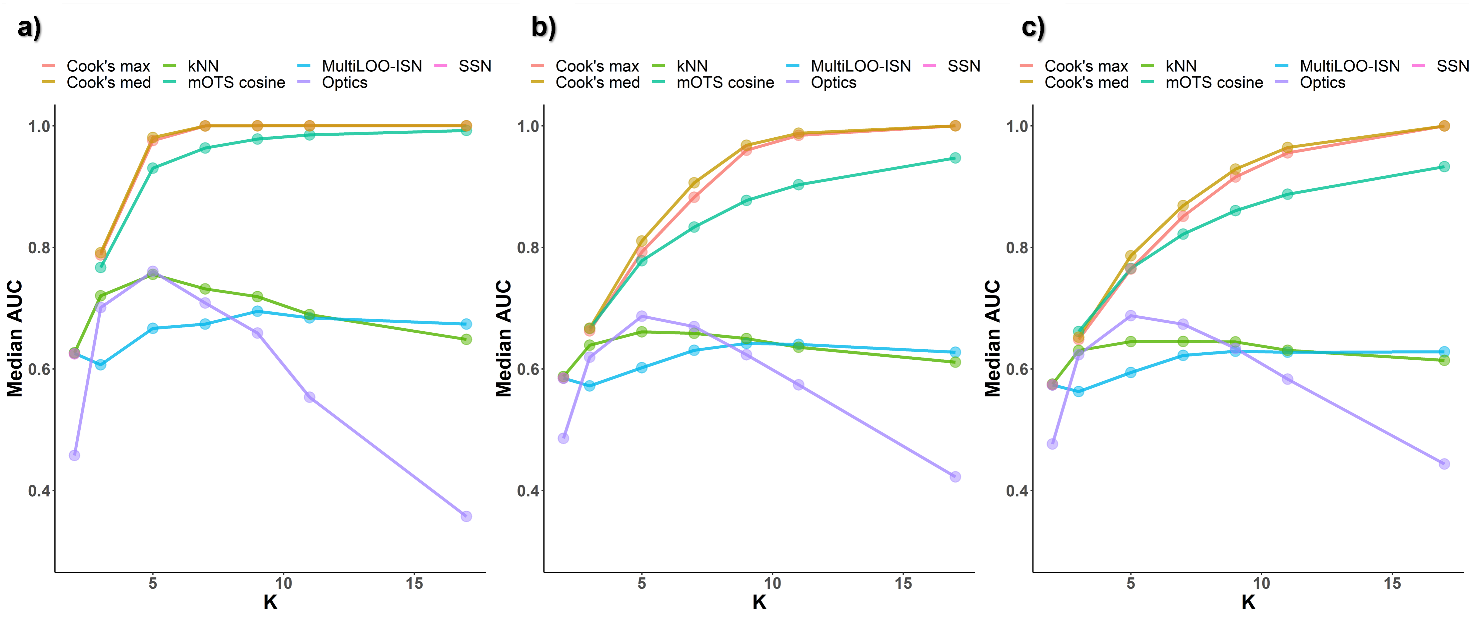
Figure **S2**: Synthetic Data: normality assumption. Median AUC values with respect to module's size *k*, for different numbers of outliers *M*, i.e., *a)* *M=1,* b) *M=5* and *c) M=10,* are depicted for all methods. The value of *AUC* slightly but consistently decreases when increasing the number of outliers *M (from left to right)*, for all methods.

**Microbiome performances varying on k and M**


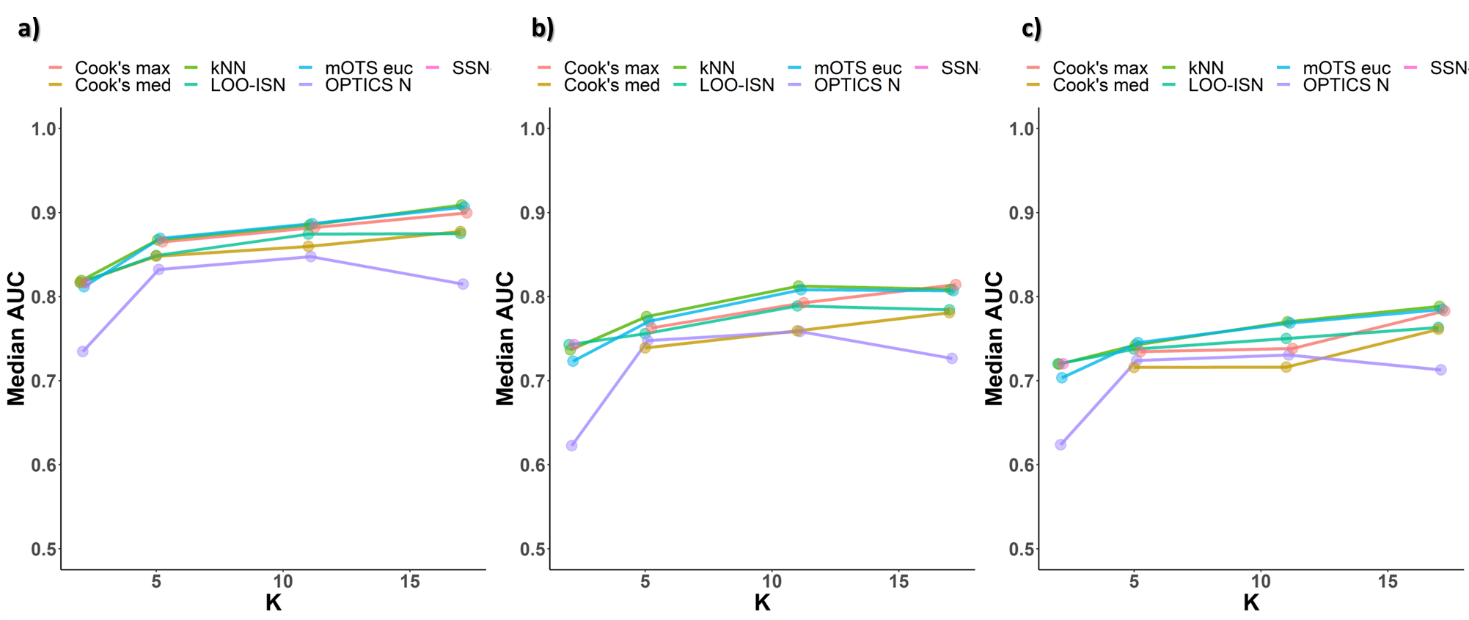


Figure **S3**: Synthetic Data: compositional. Performance of methods with respect to module's size *k* when the number of outliers M is set to *a)* *M=1,* b) *M=5* and *c) M=10*. A steady consistent decrease in performance is observed when increasing the number of outliers *M (from left to right).*

**ISN-L rationale**


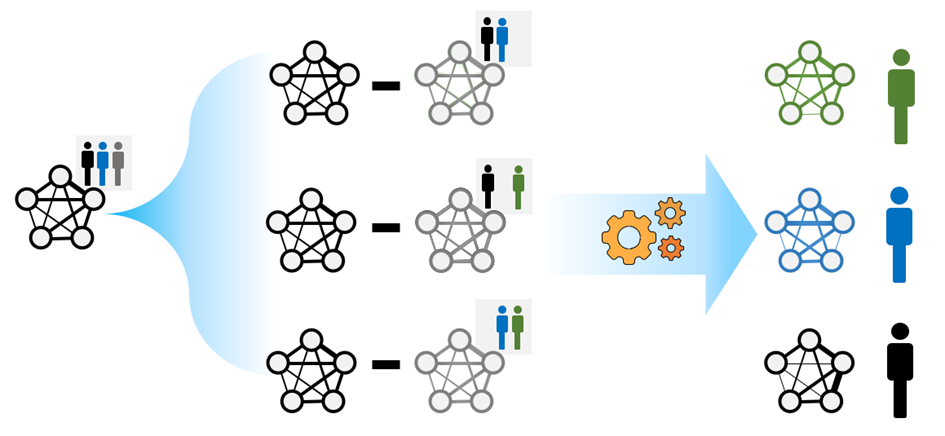


Figure **S4**: The figure depicts *ISNs-L*'s rationale. The *global* network (in black) summarises information across the population. The *LOO* network (in grey) is constructed by removing the observation *q* from the population. Hence this difference is the basis of the LIONESS procedure for computing the individual-specific network, whose specificity is in the edge weights.

**Base computation step**


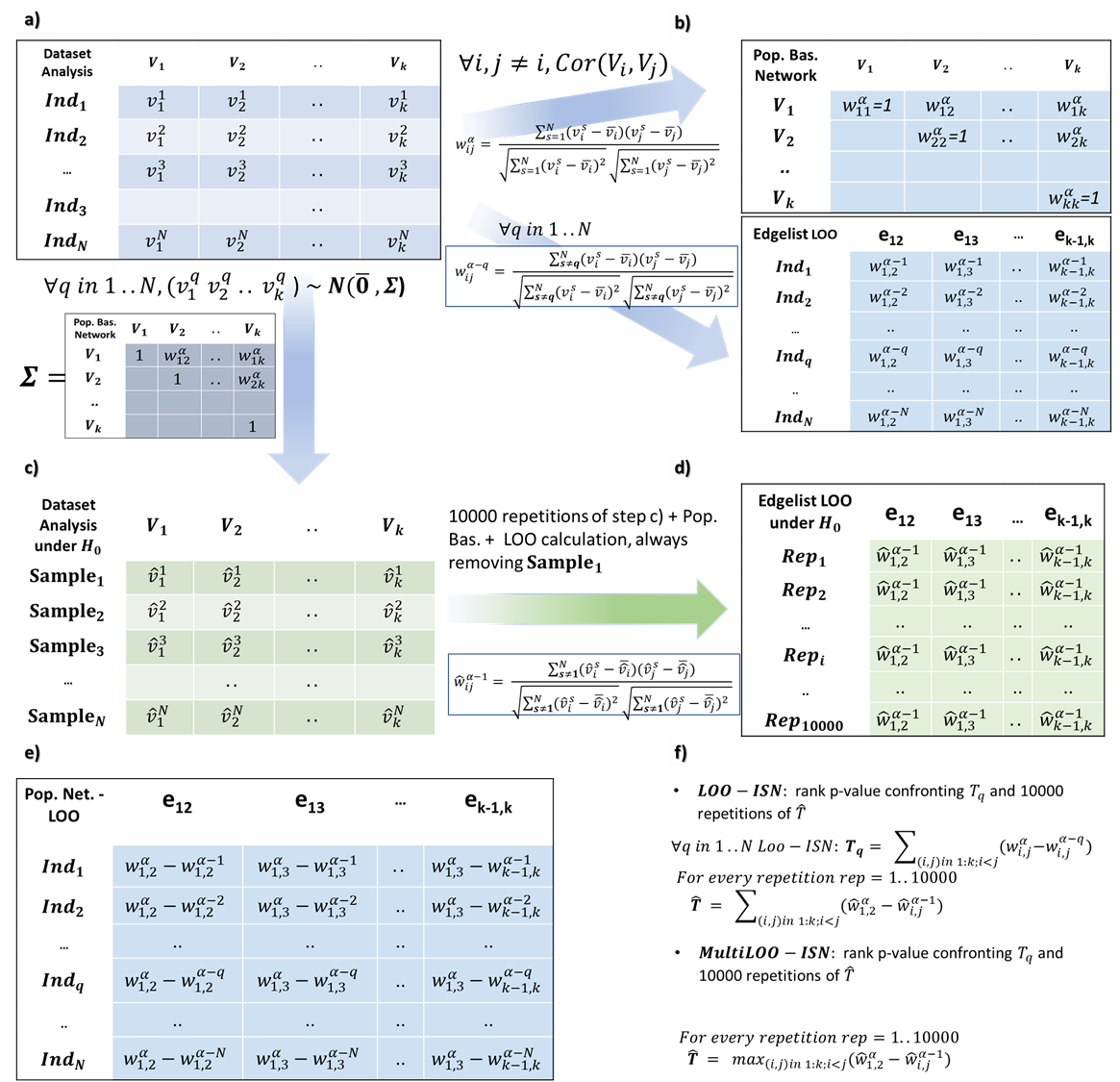


**Figure S5**: Pipeline of LOO and Multi-LOO methods. From the dataset of analysis a), we calculate the Population based network and the LOO network, here showed in the edgelist form, through Pearson correlation. Moreover, from the same dataset of analysis, we generate a dataset c) throught a multivariate normal distribution. We generate N samples – same sample size as the original dataset. In the multivariate normal we set the mean vector (dimension $kx1)$ equal to $\bar{0}$, as we suppose to work with normalized data. We set $\Sigma$, the normal matrix of covariance, equal to the calculated population-based network. Hence, this dataset is generated under the null hypothesis $H_{0}$ that each weight associated with an ISN edge is the same as its population-based counterpart. By removing one individual from the c) dataset, we calculate the LOO network under $H_{0}$. Repeating the step 10000 times d), we populate the LOO network under $H_{0}$. Finally, we compute the difference between the population-based network and the LOO in panel e). This difference is confronted with the same difference calculated under $H_{0}$ and aggregated f) with the *Max* or a *Sum* in LOO-ISN and MultiLOO-ISN.

**Data simulation pipeline: normally distributed data**


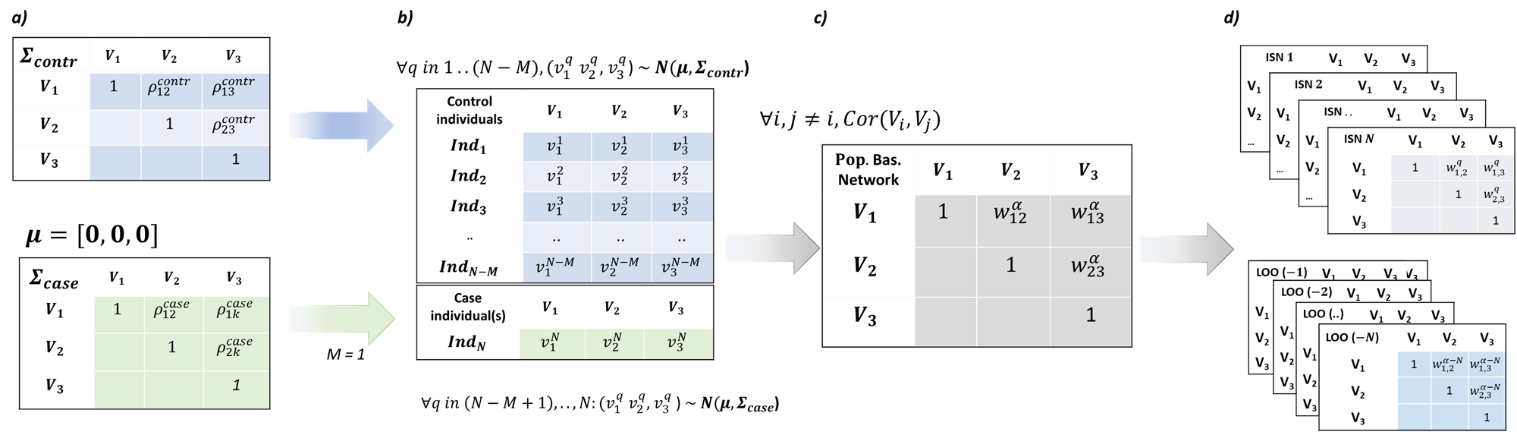


Figure **S6**: Steps to generate normally distributed data. In panel a) we generate a random correlation matrix (in R, function randcorr) for the controls $\Sigma_{\mathrm{contr}}$ and the case(s) $\Sigma_{\mathrm{case}}$. The two correlation matrices are generated independently. In this example, we set the size of the generated module to 3. Then, b) from a multivariate normal (R function mvnorm) we generate the features (nodes $v_{i}, i= 1..k$) for controls and cases(s). We used $\mu=0$as mean vector and, respectively, $\Sigma_{\mathrm{control}}$ and $\Sigma_{\mathrm{case}}$ as variance/covariance matrices for the $N-M$ controls and the$M$ case(s), 1 in our example. The simulated cases and controls are then combined to form the dataset of analysis. On the dataset of analysis, we calculate Pearson correlation c) to generate the $3x3$ population-based network. Based on this network, we calculate d) the LOO networks and the individual-specific networks. The calculated individual-specific edge weights ($w_{1,2}^{q},w_{1,3}^{q}$, and $w_{2,3}^{q}$, for an individual $q$) are used as feature set for the outlier detection methods, except for SSN-m, MultiLOO-ISN and LOO-ISN using the LOO network.

**Data simulation pipeline: compositional data**


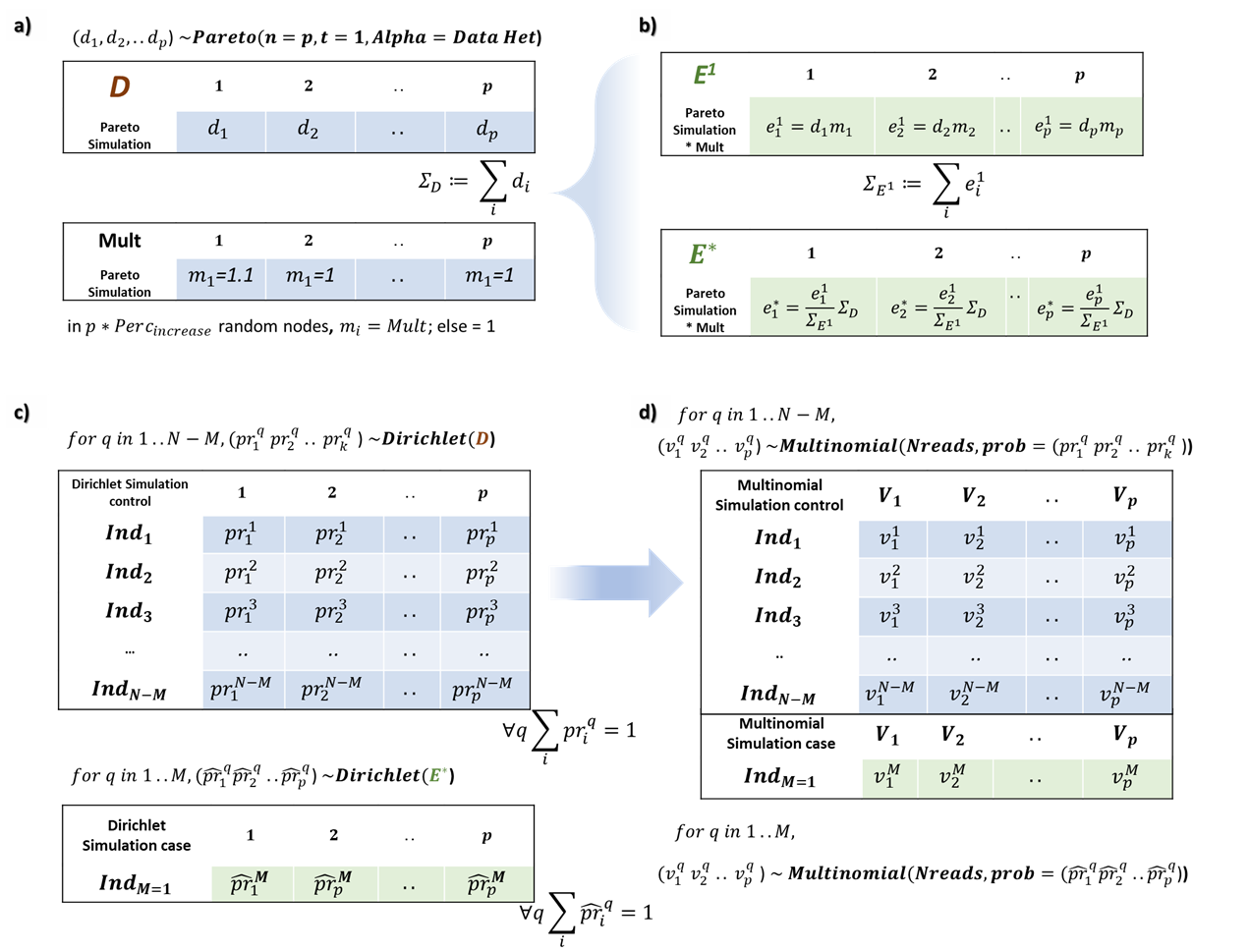


Figure **S7**: Representation of the steps to generate compositional data of dimension $Nxp$, with $p$ number of nodes and *N* the sample size. In panel a), a vector of dimension $p x1$ is generated from a Pareto function with threshold value set to $1$ and *alpha* set to our *data heterogeneity* parameter. A particular case is “No heterogeneity”, where the vector is generated from a Uniform distribution, hence $d_{i}= 1\forall i, 1..p.$ Then, on a proportion of $p$decided by the parameter *Perc increase*, the *mult* vector is set equal to the *mult* parameter ($1.1$ in the example). In panel b) we show the creation of the vector E*, the parameters to generate outlier(s) individual(s) ($1$ in the example). First, we multiply $d_{i}$ and $m_{i}$. Then, we standardized to have the same sum as D. D and E* are the parameters of probability mass used c) in a *Dirichlet* sampling to create *pr*, a personalized vector of nodes’ probability for each individual. As shown, said probability sums to *1* for a certain individual. Finally, d) *pr* is used as a parameter to generate the simulated feature, i.e., the taxa, on every individual with a multinomial. The simulated case(s) and controls are then combined to form the dataset of analysis. Said dataset is Centered-log-ratio transformed, and then the same pipeline as in panels c) and d) of Figure S6 can be applied.

**TABLES**

**TABLE S1: Glossary of Terminology.**

| **Term** | **Definition** |
| --- | --- |
| Network | In our work, a network is a set of objects, called nodes or vertices, with connections between the nodes, called edges or links. In mathematics, networks are often referred to as graphs. Hence, a network can be defined as a graph *G = (V, E),* where *V* is a set of *p* nodes and *E* is a set of *m* edges each edge being a pair *e_ij_ = (v_i_ , v_j_)*, with *v_j_ є V, i≠j, i,j=1,...,p.* Note that these classical networks are occasionally referred to as node networks to distinguish them from derived edge networks. In such an edge network, every vertex would represent an edge (pair of vertices) in the original node network. |
| Weighted network | A weighted network is a network where the connections between nodes have weights assigned to them. Using the previous mathematical description of a network, edge weights are real numbers *w_k_* k*=1,…,m.* To better show the link between the edge weight and the edge, we use the notation $w_{ij}$ for the edge weight of edge $e_{ij}.$When positive and negative weights are allowed (positive and negative associations between objects), the network is signed. Any weighted network can be sparsified into a binary network (edge is present or absent) by defining an edge weight threshold *T*. For instance, if \|*w_k_*\| *> T*, then the edge is present (*w_k_ =1)* and the edge is absent otherwise (*w_k_ =0).* For completion, edge weights are special cases of edge attributes. The edge attributes of an edge *e_ij_* in a graph *G* are represented by *r_ij_ є R^d^* , where *d* is the dimension of each edge attribute. If *d =1* then *r_ij_* is referred to as an edge weight if *r_ij_* is continuous and as an edge label if *r_ij_* is discrete. |
| Co-expression network | A co-expression network is an undirected, weighted node network with nodes corresponding to gene expression profiles. It can, therefore, also be referred to as a gene co-expression network. The edges between genes can be determined by any statistical method that captures an appropriate measure of association between genes, giving rise to weights *w_ij_* (connecting nodes $v_{i}$,$v_{j}$). In this work, we compute the Pearson correlation value between two gene expression profiles to define *w_ij._* Other examples rely on Spearman correlation or mutual information. Sometimes the association between genes is referred to as an interaction, and the gene co-expression network is called an interaction network. |
| Microbial co-occurrence network | A microbiome co-occurrence network is a node network with nodes corresponding to microbial abundance (for instance, Operational Taxonomic Units that exhibit molecular similarity are widely accepted as analytical units in microbiome research). Covariance patterns define edges and are typically used to infer microbial interactions analytically. For this reason, microbial co-occurrence networks are often coined microbial interaction networks, even though, technically, a microbial interaction is not the same as a co-occurrence and instead may point to an actual exchange or transfer of molecular and genetic information taxa. |
| Adjacency matrix | In graph theory, an adjacency matrix is a square *-p* × p matrix *A* such that its element *a_ij_* is one when there is an edge from vertex *v*_i_ to vertex *v*_j_, and zero otherwise. For a weighted network, elements *a_ij_* of an associated adjacency matrix *A* are defined as *w_ij_*, the weight *w_ij_* connecting the vertices *v*_i_ and *v_j_*, *i≠j, i,j=1,…,p.* An adjacency matrix is not unique because it depends on a numbering scheme for the vertices. An adjacency matrix can be built from any network, irrespective of whether the edges are experimentally supported or statistically inferred, and irrespective of whether it represents a network presentation of an entire population or a single individual. For completion, we mention that sometimes a function of edge weights is used to determine an adjacency matrix. For instance, a parameter *β* can be introduced such that high correlations between genes are emphasized at the expense of low correlations; in the case of a gene co-expression network this leads to *a* *_ij_* = \|cor(*gene* *_i_* , *gene_j_* )\|*^β^*  and *a* *_ij_* = \|(1 + cor(gene *_i_* , *gene_j_* ))*/* 2\|*^β^* representing the adjacency of, respectively, an unsigned and signed network. This extra parameter is handy when the aim is to identify clusters of interconnected genes in downstream analyses. |
| Module | Modules in a network are clusters of nodes and edges connecting nodes in the module. Said module can be biological, i.e., a set of nodes acting as key drivers of disease manifestation^1^. In the context of graph theory, a module can refer also to a subset of nodes that are densely connected within the subset and sparsely connected outside of the subset. For the microbiome co-occurrence networks we consider in this work, modules correspond to microbial taxa with high co-existence probability, assembling into modules of potential biological importance. |
| Network modularity | Network modularity is a measure used in network science to quantify the presence of densely interconnected subgroups, or modules, within a complex network. Higher modularity values indicate stronger division of the network into modules, making it a valuable tool for identifying functional modules, detecting community structures, and studying the structure and dynamics of complex networks. Network modularity measures the density of edges inside a subgroup/module compared to edges between subgroups. For a weighted graph, network modularity is defined as: $Q= \sum_{ij} \left( w_{ij}-\frac{k_{i}k_{j}}{2m} \right)*\delta(c_{i}c_{j})$, with $w_{ij}$ the weighted entry for the adjacency matrix, $k_{i}$ and $k_{j}$ the sum of weights for edges attached to nodes $v_{i}$ and $v_{j}$, $m$ the sum of all edge weights in the graph, $c_{i}$ and $c_{j}$ the subgroup to which the nodes belong and $\delta$the Kronecker delta function equal to 1 if ${c_{i}= c}_{j}.$ Hence, we can calculate the network modularity of a subgroup c as $Q_{c}=\frac{\Sigma_{in}}{2m}-({\frac{\Sigma_{tot}}{2m})}^{2},$ with $\Sigma_{in}$ the sum of edge weights between nodes within the subgroup (considered twice) and $\Sigma_{tot}$ the sum of all edge weights for nodes within the subgroup. |
| Normally distributed data | The Gaussian distribution, also known as the normal distribution, is a continuous probability distribution for a real-valued random variable. Its graphical representation is a bell-shaped curve. It is one of the most commonly used distributions in statistics. This is also the case for log_2_-transformed gene expression data, albeit evidence that the normality assumption does not apply uniformly to all genes in gene expression studies. When the interest is in the inference of gene correlations, a multivariate Gaussian distribution is commonly used to generate the genes. |
| Compositional data | In human microbiome studies, the absolute abundances of microbes are not recoverable from sequence data alone. Compositional data are nonnegative and capture relative information. The data are often closed because they are constrained to a constant sum (for instance, proportions adding up to 1). Such data cannot be assumed to follow a Gaussian distribution. In alternative, we use a hierarchical model combining Dirichlet and multinomial distribution. Outliers and non-outliers have different a-priori taxa distribution probabilities (sampled from a Pareto or Uniform distribution). |
| Leave-one-out | Leave-n-out procedures are common-place in statistics, in particular in cross-validation or Jacknife procedures where $n$ individuals are left out repetitively and statistics are computed on the left-out or kept samples, respectively. A special case is where *n=1*. In our work, we use the Jacknife principle to re-estimate a population network. The contrast between the original network and the reduced network (having removed a single individual) forms the basis of Kuijjer's^2^ method to construct a person-specific network. In the literature, the network specific to the removed individual is also known as individual-specific network ISN or sample-specific network SSN. |

**TABLE S2**: Characteristics of every implemented method

| *Method name* | *Principal algorithm* | *Input Network* | *Parameters* | *Aggregation technique* |
| --- | --- | --- | --- | --- |
| MultiLOO-ISN | Multivariate Leave-one-out | SSN-n | Repetitions = 20 000 | MAX in value in edges |
| LOO-ISN | Leave-one-out | SSN-n | Repetitions = 20 000 | AVG in value in edges |
| SSN-m | Leave-one-out | SSN-n | / |  |
| KNN log(N),K | kNN | ISN-L | kmin, kmax = min/max(log(N), k+1) | AVG in kmin to kmax |
| KNN log(N),K -n | kNN | SSN-n | kmin, kmax = min/max(log(N), k+1) | AVG in kmin to kmax |
| KNN 5,$\boldsymbol{\surd N}$ | kNN | ISN-L | kmin, kmax = min/max(5, √N) | AVG in kmin to kmax |
| KNN 5,$\boldsymbol{\surd N}$ -n | kNN | SSN-n | kmin, kmax = min/max(5, √N) | AVG in kmin to kmax |
| Optics avg | Optics | ISN-L | k = mean(log(N), k+1) |  |
| Optics avg -n | Optics | SSN-n | k = mean(log(N), k+1) |  |
| Optics 5 | Optics | ISN-L | k = 5 |  |
| Optics 5 -n | Optics | SSN-n | k = 5 |  |
| Optics $\boldsymbol{\surd N}$ | Optics | ISN-L | k = √N |  |
| Optics $\boldsymbol{\surd N}$ -n | Optics | SSN-n | k = √N |  |
| OTS euclidean | Spoutlier | ISN-L | refs = 20 |  |
| OTS euclidean -n | Spoutlier | SSN-n | refs = 20 |  |
| OTS cosine | Spoutlier | ISN-L | refs = 20 |  |
| OTS cosine -n | Spoutlier | SSN-n | refs = 20 |  |
| mOTS cosine | Spoutlier | ISN-L | refs = 20 | MED in cosine 20 runs |
| mOTS euc | Spoutlier | ISN-L | refs = 20 | MED in euclidean 20 runs |
| mOTS glob | Spoutlier | ISN-L | refs = 20 | MED in all 20+20 runs |
| Spoutlier | Spoutlier | SSN-n | refs = 20 | / |
| Spoutlier -n | Spoutlier | SSN-n | refs = 20 | / |
| Spoutlier -n norm | Spoutlier | SSN-n | refs = 20 | / |
| Cook's max | Cook's distance | ISN-L | / | MAX cook in all runs |
| Cook's max -n | Cook's distance | SSN-n | / | MAX cook in all runs |
| Cook's avg | Cook's distance | ISN-L | / | AVG cook in all runs |
| Cook's avg -n | Cook's distance | SSN-n | / | AVG cook in all runs |
| Cook's med | Cook's distance | ISN-L | / | MED cook in all runs |
| Cook's med -n | Cook's distance | SSN-n | / | MED cook in all runs |

**TABLE S3:** Averaged *AUC* results for each instance of each method. This coarse summarization yields a first impression of the most effective techniques.

| *Method* | *Median AUC* | *Mean AUC* |
| --- | --- | --- |
| MultiLOO-ISN | 0.628 | 0.632 |
| LOO-ISN | 0.582 | 0.591 |
| SSN-m | 0.584 | 0.601 |
| KNN log(N),K | 0.646 | 0.657 |
| KNN log(N),K -n | 0.646 | 0.657 |
| KNN 5,$\boldsymbol{\surd N}$ | 0.649 | 0.659 |
| KNN 5,$\boldsymbol{\surd N}$ -n | 0.649 | 0.659 |
| Optics avg | 0.510 | 0.529 |
| Optics avg -n | 0.510 | 0.529 |
| Optics 5 | 0.608 | 0.595 |
| Optics 5 -n | 0.608 | 0.595 |
| Optics $\boldsymbol{\surd N}$ | 0.532 | 0.529 |
| Optics $\boldsymbol{\surd N}$ -n | 0.532 | 0.529 |
| OTS euclidean | 0.628 | 0.637 |
| OTS euclidean -n | 0.632 | 0.640 |
| OTS cosine | 0.812 | 0.773 |
| OTS cosine -n | 0.739 | 0.708 |
| mOTS cosine | 0.880 | **0.866** |
| mOTS euc | 0.629 | 0.640 |
| mOTS glob | 0.820 | 0.824 |
| Spoutlier | 0.628 | 0.639 |
| Spoutlier -n | 0.631 | 0.639 |
| Spoutlier -n norm | 0.616 | 0.658 |
| Cook's max | 0.903 | 0.853 |
| Cook's max -n | 0.903 | 0.853 |
| Cook's avg | 0.917 | 0.858 |
| Cook's avg -n | 0.917 | 0.858 |
| Cook's med | **0.920** | 0.859 |
| Cook's med -n | **0.920** | 0.859 |

**TABLE S4:** Averaged *AUC* for each instance of the context of *high* heterogeneity and *elevate* multiplier in synthetic data.

| Mult 2 & Pareto - 0.7 | | |  |
| --- | --- | --- | --- |
| *Method* | ***Median AUC*** | ***Mean AUC*** | |
| MultiLOO-ISN | 0.780 | 0.794 | |
| LOO-ISN | 0.788 | 0.788 | |
| SSN-m | 0.758 | 0.760 | |
| KNN log(N),K | 0.800 | 0.800 | |
| KNN log(N),K -n | 0.800 | 0.800 | |
| KNN 5, $\boldsymbol{\surd N}$ | **0.801** | **0.803** | |
| KNN 5, $\boldsymbol{\surd N}$ -n | **0.801** | **0.803** | |
| Optics avg | 0.697 | 0.685 | |
| Optics avg -n | 0.697 | 0.685 | |
| Optics 5 | 0.686 | 0.669 | |
| Optics 5 -n | 0.686 | 0.669 | |
| Optics $\boldsymbol{\surd N}$ | 0.739 | 0.740 | |
| Optics $\boldsymbol{\surd N}$ -n | 0.739 | 0.740 | |
| OTS euclidean | 0.786 | 0.786 | |
| OTS euclidean -n | 0.786 | 0.788 | |
| OTS cosine | 0.515 | 0.519 | |
| OTS cosine -n | 0.517 | 0.522 | |
| mOTS cosine | 0.542 | 0.544 | |
| mOTS euc | 0.800 | 0.799 | |
| mOTS glob | 0.739 | 0.754 | |
| Spoutlier | 0.789 | 0.787 | |
| Spoutlier -n | 0.786 | 0.788 | |
| Spoutlier -n norm | 0.783 | 0.786 | |
| Cook's max | 0.786 | 0.793 | |
| Cook's max -n | 0.786 | 0.793 | |
| Cook's avg | 0.783 | 0.789 | |
| Cook's avg -n | 0.783 | 0.789 | |
| Cook's med | 0.775 | 0.776 | |
| Cook's med -n | 0.775 | 0.775 | |

**TABLE S5**: Summarization of all methods of each family coarse *Median* and *Mean* performances per *Mult* parameter - if the average abundances for the *cases* observation is 10%, 50%, 100% more - and *Data* *Heterogeneity* - from null to mild and high. High multipliers and more heterogeneity yield better *AUC*. With a *Mult* of 1.1, there is no value in outlier detection: it is not better than random guessing.

| Method | MULTIPLIER 1.1 | | MULTIPLIER 1.5 | | MULTIPLIER 2.0 | | UNIFORM –  NO HET | | PARETO -  4  MILD HET | | PARETO - 0.7 HIGH HET | |
| --- | --- | --- | --- | --- | --- | --- | --- | --- | --- | --- | --- | --- |
|  | ***Median AUC*** | ***Mean AUC*** | ***Median AUC*** | ***Mean AUC*** | ***Median AUC*** | ***Mean AUC*** | ***Median AUC*** | ***Mean AUC*** | ***Median AUC*** | ***Mean AUC*** | ***Median AUC*** | ***Mean AUC*** |
| MultiLOO-ISN | 0.502 | 0.502 | 0.565 | 0.586 | 0.684 | 0.705 | 0.543 | 0.562 | 0.556 | 0.578 | 0.629 | 0.653 |
| LOO-ISN | 0.503 | **0.505** | **0.586** | **0.598** | **0.726** | **0.733** | **0.559** | **0.585** | **0.571** | **0.601** | 0.637 | 0.650 |
| SSN - m | 0.498 | 0.497 | 0.559 | 0.577 | 0.676 | 0.695 | 0.541 | 0.562 | 0.553 | 0.575 | 0.619 | 0.632 |
| KNN log(N),P | 0.503 | 0.503 | 0.582 | 0.596 | 0.714 | 0.730 | 0.556 | 0.579 | 0.567 | 0.595 | 0.642 | 0.656 |
| KNN log(N),P -n | 0.503 | 0.503 | 0.582 | 0.596 | 0.714 | 0.730 | 0.556 | 0.579 | 0.567 | 0.595 | 0.642 | 0.656 |
| KNN 5, $\boldsymbol{\surd N}$ | **0.504** | 0.502 | 0.582 | 0.597 | 0.717 | 0.732 | 0.556 | 0.579 | 0.568 | 0.595 | **0.644** | **0.657** |
| KNN 5, $\boldsymbol{\surd N}$ -n | **0.504** | 0.502 | 0.582 | 0.597 | 0.717 | 0.732 | 0.556 | 0.579 | 0.568 | 0.595 | **0.644** | **0.657** |
| Optics avg | 0.483 | 0.459 | 0.540 | 0.527 | 0.636 | 0.635 | 0.501 | 0.518 | 0.511 | 0.531 | 0.527 | 0.572 |
| Optics avg -n | 0.483 | 0.459 | 0.540 | 0.527 | 0.636 | 0.635 | 0.501 | 0.518 | 0.511 | 0.531 | 0.527 | 0.572 |
| Optics 5 | 0.494 | 0.477 | 0.541 | 0.533 | 0.633 | 0.624 | 0.510 | 0.525 | 0.521 | 0.536 | 0.546 | 0.573 |
| Optics 5 -n | 0.494 | 0.477 | 0.541 | 0.533 | 0.633 | 0.624 | 0.510 | 0.525 | 0.521 | 0.536 | 0.546 | 0.573 |
| Optics $\boldsymbol{\surd N}$ | 0.491 | 0.489 | 0.556 | 0.565 | 0.673 | 0.686 | 0.532 | 0.555 | 0.545 | 0.569 | 0.595 | 0.616 |
| Optics $\boldsymbol{\surd N}$ -n | 0.491 | 0.489 | 0.556 | 0.565 | 0.673 | 0.686 | 0.532 | 0.555 | 0.545 | 0.569 | 0.595 | 0.616 |
| OTS | 0.503 | 0.503 | 0.577 | 0.591 | 0.704 | 0.718 | 0.553 | 0.574 | 0.566 | 0.590 | 0.635 | 0.648 |
| OTS -n | 0.503 | 0.503 | 0.577 | 0.591 | 0.705 | 0.719 | 0.552 | 0.574 | 0.564 | 0.591 | 0.632 | 0.648 |
| OTS cosine | 0.499 | 0.500 | 0.504 | 0.504 | 0.499 | 0.503 | 0.498 | 0.497 | 0.496 | 0.495 | 0.509 | 0.514 |
| OTS cosine -n | 0.500 | 0.502 | 0.501 | 0.499 | 0.501 | 0.504 | 0.497 | 0.497 | 0.498 | 0.496 | 0.510 | 0.512 |
| mOTS cosine | 0.499 | 0.499 | 0.503 | 0.506 | 0.497 | 0.505 | 0.494 | 0.493 | 0.491 | 0.491 | 0.519 | 0.525 |
| mOTS euc | **0.504** | 0.503 | 0.581 | 0.595 | 0.714 | 0.729 | 0.554 | 0.578 | 0.569 | 0.595 | 0.637 | 0.655 |
| mOTS glob | 0.502 | 0.503 | 0.551 | 0.571 | 0.642 | 0.672 | 0.532 | 0.550 | 0.547 | 0.564 | 0.614 | 0.631 |
| Spoutlier | 0.503 | 0.501 | 0.576 | 0.590 | 0.703 | 0.720 | 0.551 | 0.575 | 0.561 | 0.589 | 0.634 | 0.646 |
| Spoutlier -n | 0.501 | 0.501 | 0.575 | 0.590 | 0.704 | 0.719 | 0.552 | 0.573 | 0.564 | 0.590 | 0.627 | 0.648 |
| Spoutlier -n norm | 0.501 | 0.501 | 0.576 | 0.591 | 0.706 | 0.720 | 0.557 | 0.574 | 0.562 | 0.591 | 0.634 | 0.647 |
| Cook's max | 0.501 | 0.502 | 0.575 | 0.594 | 0.703 | 0.720 | 0.554 | 0.574 | 0.564 | 0.589 | 0.637 | 0.652 |
| Cook's max -n | 0.501 | 0.502 | 0.575 | 0.594 | 0.703 | 0.720 | 0.554 | 0.574 | 0.564 | 0.589 | 0.637 | 0.652 |
| Cook's avg | 0.502 | 0.502 | 0.581 | 0.596 | 0.713 | 0.723 | 0.555 | 0.578 | 0.563 | 0.593 | 0.640 | 0.650 |
| Cook's avg -n | 0.502 | 0.502 | 0.581 | 0.596 | 0.713 | 0.723 | 0.555 | 0.578 | 0.563 | 0.593 | 0.640 | 0.650 |
| Cook's med | 0.502 | 0.503 | 0.580 | 0.593 | 0.712 | 0.718 | 0.555 | 0.578 | 0.566 | 0.592 | 0.630 | 0.644 |
| Cook's med -n | 0.502 | 0.503 | 0.580 | 0.593 | 0.712 | 0.718 | 0.555 | 0.578 | 0.566 | 0.592 | 0.630 | 0.644 |

**Methods**

### LOO-ISN

Pseudocode for the LOO-ISN algorithm.


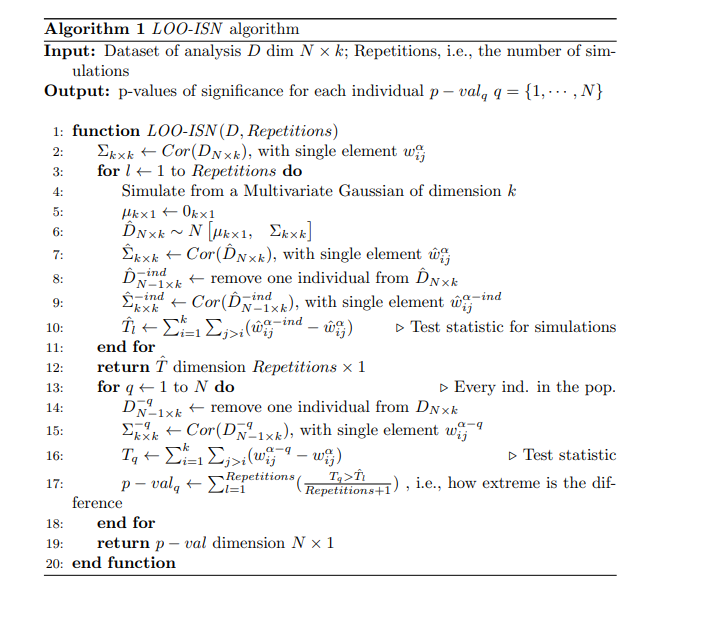


### MultiLOO-ISN

Pseudocode for the MultiLOO-ISN algorithm.


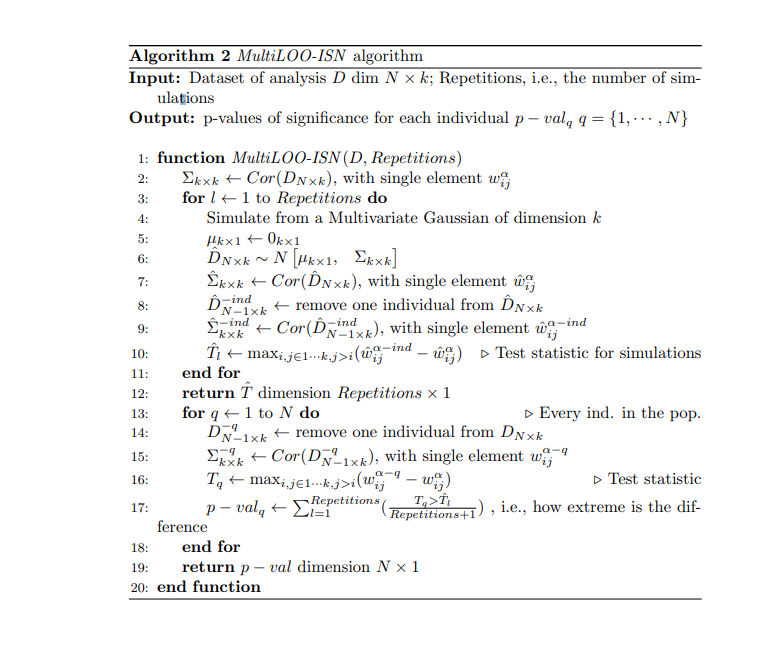


Marked differences with the *LOO-ISN* pseudocode of the previous paragraph are lines 10 and 16, where the edge weight aggregation is done with the max() and not with the average

### kNN

*kNN*’s rationale is that an observation shares similarities and patterns with close observations. Hence, we can exploit the phenotype of those *neighbors* to classify the target. The main parameter $k$ defines the number of *neighbors* considered for every target observation. Notably, we focus on the implementation from Angiulli. This extension has been developed for outlier detection. Angiulli’s *kNN* averages results around a set of $k$ values, with $k_{min}$ and a $k_{max}$ as extremes.

We took the individual edge weights belonging to a module $Me=\{w_{ij}^{q}:i,j\in M_{d}\}$ as features to calculate a Euclidean distance between an observation and its $k$ nearest neighbors. We explored multiple sets of $k_{min}$ and $k_{max}$. The distance between two individuals *q* and *s* is:

$$d(q,s)=\sqrt{\sum_{(i,j)\in Md} (w_{ij}^{q}-w_{ij}^{s})^{2}}$$

$d(q,s)$ is then averaged across the *k*-neighbors of *q* to find the *OS* of individual *q*.
*kNN*, as well as *OPTICS, Spoutlier* and *Cook’s distance*, does not return a p-value but an outlier score *OS* for each observation. For a given module, this score is assigned to every individual and can be ranked to find a hierarchy of individuals keener to be outliers.

### DBSCAN - OPTICS

*DBSCAN* is a density-based approach using a notion of density-reachability that classifies points into three main categories: core points, border points and noise points. Core points are the ones that have at least $minPts$ (a parameter) points into their radius $\epsilon$ (another parameter), defining the $\epsilon-$Neighborhood $N_{\epsilon}(p)$. Border points are points into $\epsilon$ distance from at least one core point, while noise points are unreachable from a core point. The base *DBSCAN* method is inadequate for the outlier detection goal; hence, the *OPTICS-OF* (simply referred as OPTICS in the paper) enhancement is used. The base assumption is that being an outlier is not a binary condition but a property appliable, with different degrees, to all the points in a dataset. Focal points are the concept of Core-distance and Reachability-Distance.

Core-distance ($core\_dist$) is the minimum distance $\epsilon$ such that the observation $q$ can not be defined as a core point with respect to $\epsilon$ and $MinPts$. This distance can be undefined if there is no $\epsilon$ such that $q$ can be a core point.

$$core\_dist_{\epsilon,MinPts}(p)=\left\{ \begin{matrix} \text{Undefined} & \text{if }|N_{\varepsilon}(p)|<MinPts \\ MinPts\text{-th smallest distance in }N_{\varepsilon}(p) & \text{otherwise} \end{matrix} \right.$$

The reachability-distance ($reachability\_dist$) of another point *o* from a point *p* is either the distance between *o* and *p*, or the core distance of *p*, whichever is bigger. It is the minimum distance measure such that $p$ is directly density reachable from $o$, if $o$ is a core point in the $\epsilon$ -neighborhood of $q$. Such distance can never be lower than core-distance and can also be undefined if there is no $eps$ such that $q$ can be a core point.

$$reachability_{dist_{\epsilon,MinPts}\left( o,p \right)}=\left\{ \begin{matrix} \text{Undefined} & \text{if }\left| N_{\varepsilon}\left( p \right) \right|<MinPts \\ \text{max}\left( \text{core\_dist}_{\varepsilon,MinPts}\left( p \right),\text{dist}\left( p,o \right) \right) & \text{otherwise} \end{matrix} \right.$$

Local-reachability distance ($lrd$) is the inverse of the mean reachability distance of the closer $MinPt$s. For this formula, all the reachability distance needs to be defined, so we should set an adequately high $\epsilon$.

$${lrd}_{\epsilon,MinPts}\left( p \right)=1 / \left( \frac{\sum_{o\in N_{\epsilon}(p)} {reachability\_dist}_{\epsilon,MinPts}(o,p)}{\left| N_{\epsilon}(p) \right|} \right)$$

Outlier factor ($OF$) is calculated on the basis of local-reachability distance. $OF$ defines the outlier degree for each individual.

$${OF}_{\epsilon,MinPts}\left( p \right)= \frac{\sum_{o\in N_{\epsilon}(p)} \frac{{lrd}_{\epsilon,MinPts}(o)}{{lrd}_{\epsilon,MinPts}(p)}}{\left| N_{\epsilon}(p) \right|}$$

## **Software analysis and visualization**

In this work, several different visualization strategies have been applied. To nicely highlight the core insights emerged, the basic approach is to choose between all the various methods of a family (varying parameters, inputs, inner function.. ) the most representative ones, strongly related to the ones yielding better performance, and represent them in a plot. Given that the settings considered are highly heterogeneous, the visualization goal is also to yield insights regarding the performance heterogeneity varying parameters. Particularly, sample size and module’s size are of special interest and the graphs will aim to highlight their peculiarity. That allows to carefully design the experiment beforehand, considering the specific sample size values and understanding the influence of a modularity framework over significance.

The whole analysis was carried in R version 4.0.3 and tested both on a $x86\_64-w64-mingw32/x64$ and on the server of University of Liége. The code is available upon request. For the graphs visualization, we used the libraries *ggplot2* and *igraph*. The methods rely on the packages *spoutlier*, *randcorr*, *dbscan*, *pROC*, *DDOutlier* and *lsa*. The packages employed for the microbiome data simulations are *gtools* and *Pareto*. The compositional and gaussian simulations and evaluated were performed in the Liége cluster.

**Community detection - SPINGLASS**

Between the many available community detection algorithms, we decided to use *SPINGLASS*. Indeed, we excluded the Louvain algorithm, that despite its speed and precision, is not suited for our goal; i.e., it is restricted to networks with positive weight values.

Furthermore, we excluded binarization and distribution-based transformations to avoid selecting an arbitrary threshold and to avoid applying a potentially biasing transformation to the data. The *SPINGLASS* algorithm also has the advantage of being tunable through the parameter α, which allows modulating the level of granularity, i.e., the clusters' dimensions, of the recovered modules. The parameters (γ number *of spins*, i.e., the upper limit for the number of modules, *cooling factor*, *start temperature* and *stop temperature* are set to their default values except for *stop temperature,* which is set to *0.001* to increase the algorithm's granularity.

## **Graph filtration curve**

Graph filtration curves are graph representations that can be applied to labeled and unlabeled datasets, using the graph's relevant attributes and structural information. An increasing threshold is considered, and those *edges* whose weight happens to be smaller than the current threshold value are zeroed-out. A metric summarizing the subgraph is then calculated for different values of the threshold. In more detail, we use the algebraic connectivity of graphs, the so-called Fiedler value. This metric is the second smallest eigenvalue of the Laplacian matrix of a graph, and it measures how well a graph is connected; the larger the *Fiedler value*, the stronger the connectivity of the graph.

Filtration curves are applied on datasets in which two group - usually *cases* and *controls* - exist. in this work, we divided the data into (i) the putative outlier and (2) an *Average*, a group formed by all but the alleged outlier. We calculated a confidence interval for the *Average* group, by addings/substracting the standard deviation. The generated graphs, for every individual as outlier, are available in the Supplementary.

**Results**

## **Results on Real-Life Data - The LucKi Gut subcohort**

Microbiome co-occurrence networks are known to be rich in terms of the information they contain about the health conditions of individuals. Hence, we use data from the LucKi Gut cohort, an ongoing study that monitors gut microbiota development throughout infancy and early childhood, to validate the findings. We focus on 81 individuals with microbial profile available at 6 month after birth. After a prevalence ($<10\%$) step, 126 taxa survived. We CLR-transform the data and compute the Pearson correlation network on the whole dataset. Modules are discovered starting from the clusters obtained by applying the community detection algorithm *SPINGLASS* on the global network, i.e., the network computed with all the individuals. We found $4$ microbiotic modules of dimension {45,41,35,5} taxa.

Here, we analyze modules 1-3, complementing the analysis in the main text. We applied outstanding techniques from synthetic data on the *ISN-l* of module $1-3$. In particular, we apply *kNN 5* $\sqrt{N}$, *mOTS euc*, *MultiLOO-ISN*, *LOO-ISN* *mOTS cosine* and *mOTS cosine -n.* There are more features (edges) than observations (individuals), hence *Cook’s max* can not be applied.

**Module 1**

On module 1 *MultiLOO-ISN*, *LOO-ISN* found respectively $45$ and $0$ outliers. We apply rank aggregation to create an ensemble ranking of the observations. We consider the average between *MultiLOO-ISN’s* and *LOO-ISN’s* number of outliers to choose the top-k (top 22 in this case). We do not find any enrichment for external phenotypes mode of delivery or diet. The top 22 significant outliers for *MultiLOO-ISN* are non-independent distributed, i.e., Fisher exact test, testing the null hypothesis of independence of rows and columns in a contingency table with fixed marginal, with respect to the *DMM* clusters (p-value 0.015). Enrichment FDR-corrected p-value for DMM cluster 2 is 0.052.

**Module 2**

On module 2 *MultiLOO-ISN*, *LOO-ISN* found respectively $44$ and $2$ significant outliers. We apply rank aggregation to create an ensemble ranking of the observations. We consider top 23 as the average between *MultiLOO-ISN’s* and *LOO-ISN’s* for the number of outliers. We do not find any enrichment for external phenotypes mode of delivery or diet. Moreover, the taxa composing module 1 belong to phyla. Module 2’s microbes are not independently distributed with respect to the phyla of the 126 starting microbes (Fisher exact test, p-value = 1.3*10^-3). We do not find enrichment for any phyla.

**Module 3**

On module 3 *MultiLOO-ISN*, *LOO-ISN* found respectively $37$ and $5$ significant outliers. Hence, we consider the top 21 from the ensemble ranking. After multiple test correction (FDR), we did not find significance for mode of delivery, i.e., if the newborn delivery was C-section or Vaginal or diet. Although, we found non-indipendent distribution, as for the Fisher exact test, for the comparison between the top-21 outliers vs the *DMM cluster (p-value = 0.012). Module’s 3 microbes are enriched for the Proteobacteria phylum (FDR-corrected hypergeometric p-value = 2.10*10^-6).*

To synthetize, *MultiLOO-ISN*, especially in high-dimensional module, has a less stringent threshold than *LOO-ISN.* This is clear given the two different rationales. It became more and more likely to find at least an edge extreme (*MultiLOO-ISN* definition) than to find the entire module extreme (*LOO-ISN* definition*).* The limited sample size does not help the significance assessment but nonetheless we found quite some variety in the modules. Moreover, given the module’s characteristics, we know who the outliers are. This is helpful where we know the module is associated with a disease or drug response, and we want to know for who the treatment might be beneficial.

**Discussion**

## **Graph filtration curve**

The exceptional nature of the identified outliers cannot be suspected by looking at the full ISN. Novel visualization of ISNs may be better suited to further contribute to their integration in biomedical applications. We have used graph filtration curves. Distance measures can be defined in the corresponding vector space, as suggested by O’bray. Moreover, even though the presented graphs only highlight one characteristic of the network (here the Fiedler value), in principle, they can be extended to multiple dimensions covering multiple network characteristics.

## **Comparing SSN-n and ISN networks**

In the presented work, networks generated from LIONESS (ISNs-L) and SSN-n are implemented. That choice is justified by the vast popularity of those methods in the individual network field. Moreover, both of them rely on the difference between a global and a *LOO* network, quantifying the impact of an individual in the global estimate. That clearly collocates those methods in the same area and encourages the comparative use of them in the same settings.

As shown in Table [1](#3dy6vkm) and Tables S2-4, there is little difference between *ISNs* and *SSN-n*. When we compare the performances obtained with the same method and the same characteristics, e.g., KNN *5,sqrt(N)*, the performances are similar. In particular, the only case in which the $AUC$ performance differs for more than $0.05$ is *OTS cosine* under normality assumption (Table [1](#3dy6vkm)).

The similarity in the performances, as highlighted in the discussion, is due to the shared core of *SSN-n* and *ISN*. *SSN-n* and *ISN* diverge in terms of the interpretation.

It is important to note that, as specified in the main text, *SSN-m*, *LOO-ISN* and *MultiLOO-ISN* are, by their characteristics, only computed confronting population-based and LOO network. Hence, they can not be apply on ISNs-L but only on *SSN-n*.

**Comparing reference set methods in Spoutlier**

As highlighted from comparison in Tables 1-2 and S2-4 for Spoutlier and OTS entries, No notable difference emerges between the custom ($s+1$, OTS) and the literature ($s$, Spoutlier) definition of the reference set. This is expected given that the only situation in which a difference is expected - low variability, high sample size, discrete features - is not considered. It may constitute the object of future research.

## **All methods characteristics, combinations and approaches**

Multiple methods, with multiple parameters and characteristics are used. We decided to provide a throughout description of every choice in the Supplementary to preserve the text’s flow. In Table [S1](#1t3h5sf) we show the characteristics in detail.

For *kNN* family, we implemented $4$ different methods. $KNN$ $log(N),P$, $KNN$ $log(N),P-n$, $KNN$ $5,\sqrt{N}$ and $KNN$ $5,\sqrt{N}-n$. The first two sets $k_{min}$ and $k_{max}$ to minimum and maximum among the $log$ of the sample size and the number of features. Hence, we considered jointly the feature and the sample dimension. The $-n$ part at the end indicates that the calculation was on the $SSN-n$ network. $KNN$ $5,\sqrt{N}$ and $KNN$ $5,\sqrt{N}-n$ sets the $k_{min}$ and $k{}_{max}$ parameters to the minimum/maximum of 5 and the square root of the number of samples. $5$ is often used as a rule of thumb in $kNN$ and the $\sqrt{n}$ takes into account the sample size.

For *Optics* family, we implemented $6$ different methods, varying the parameter $k$. $Optics$ $avg$,$Optics$ $avg-n$ $Optics$ $\sqrt{N}$, $Optics$ $\sqrt{N}-n$, $Optics$ $5$ and $Optics$ $5-n$.
$Optics$ $avg$ sets the parameter $k$ to the average between the number of features and the logaritm of the sample size.$Optics$ $5$ and $Optics$ $\sqrt{N}$ set $k$ respectively to $5$ and the square root of $N$. We observe (Table [1](#3dy6vkm)) that $k=5$ yield the best results under normality assumption, while $k=\sqrt{n}$ is the best in compositional data (Table [2](#4d34og8) and Tables S3-4).

For *Spoutlier* family, we implemented $10$ different methods, varying the aggregation function, the reference set and the distance measure: *OTS euclidean, OTS euclidean -n, OTS cosine, OTS cosine -n, mOTS euc, mOTS cosine, mOTS glob, Spoutlier, Spoutlier -n, Spoutlier -n norm. OTS euclidean, OTS euclidean -n, mOTS euc, Spoutlier, Spoutlier -n, Spoutlier -n norm* use the euclidean distance measure, while *OTS cosine, OTS cosine -n and mOTS cosine* use a measure of cosine similarity*.* The prefix m referred to having an ensemble in which 10 different run are averaged. Spoutlier methods have the original reference set, i.e., selecting *s* observations and discarding eventual distances equal to 0. OTS methods implement the novel reference set, in which s+1 observation are taken and s (excluding the target one) are used to compute the distance. *Spoutlier -n norm* refers to a normalization of the data prior to the method, as advised by the authors. No relevant differences are found. We also observe no relevant variation between the two reference set methods. Euclidean methods are more performant in Transcriptomic simulations, while cosine similarity performs better in Microbiome simulations.

For *Cook’s distance* family, we implemented 6 different methods, varying the aggregation function: *Cook’s max, Cook’s max -n, Cook’s avg, Cook’s avg -n, Cook’s med, Cook’s med -n.* For every edge in a module, we build a linear regression, and we predict with the other edge weights in the module. Hence, we calculate the Cook’s distance. We repeat this step for every edge as target. Hence, for each edge weight we have a collection of Cook’s distance. To find the final Outlier score, we aggregate them using (1) the max (i.e., Cook’s max and Cook’s max -n); (2) the mean (*Cook’s avg, Cook’s avg -n), (3)* the median *(i.e., Cook’s med, Cook’s med -n.* ).

**Bibliography**

1. Gregorich, M. *et al.* Individual-specific networks for prediction modelling - A scoping review of methods. *BMC Med. Res. Methodol.* **22**, (2022).

2. Kuijjer, M. L., Tung, M. G., Yuan, G., Quackenbush, J. & Glass, K. Estimating Sample-Specific Regulatory Networks. *iScience* **14**, 226–240 (2019).
